# Extending the Range of Distances Accessible by ^19^F Electron–Nuclear Double Resonance in Proteins Using High-Spin Gd(III) Labels

**DOI:** 10.1021/jacs.3c13745

**Published:** 2024-02-23

**Authors:** Alexey Bogdanov, Veronica Frydman, Manas Seal, Leonid Rapatskiy, Alexander Schnegg, Wenkai Zhu, Mark Iron, Angela M. Gronenborn, Daniella Goldfarb

**Affiliations:** † Department of Chemical and Biological Physics, The Weizmann Institute of Science, P.O. Box 26, Rehovot, 7610001, Israel; ‡ Department of Chemical Research Support, The Weizmann Institute of Science, P.O. Box 26, Rehovot, 7610001, Israel; § Max Planck Institute for Chemical Energy Conversion, 34-36 Stiftstraße, Mülheim an der Ruhr, 45470, Germany; ∥ Department of Structural Biology, University of Pittsburgh, 4200 Fifth Avenue, Pittsburgh, Pennsylvania 15260, United States

## Abstract

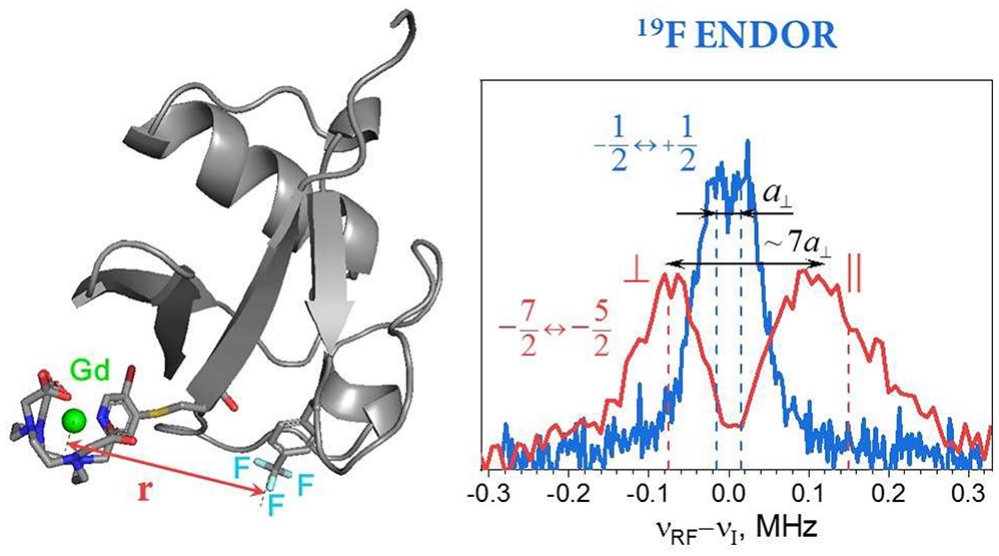

Fluorine electron-nuclear double resonance (^19^F ENDOR) has recently emerged as a valuable tool in structural biology for distance determination between F atoms and a paramagnetic center, either intrinsic or conjugated to a biomolecule via spin labeling. Such measurements allow access to distances too short to be measured by double electron–electron resonance (DEER). To further extend the accessible distance range, we exploit the high-spin properties of Gd(III) and focus on transitions other than the central transition (|−1/2⟩ ↔ |+1/2⟩), that become more populated at high magnetic fields and low temperatures. This increases the spectral resolution up to ca. 7 times, thus raising the long-distance limit of ^19^F ENDOR almost 2-fold. We first demonstrate this on a model fluorine-containing Gd(III) complex with a well-resolved ^19^F spectrum in conventional central transition measurements and show quantitative agreement between the experimental spectra and theoretical predictions. We then validate our approach on two proteins labeled with ^19^F and Gd(III), in which the Gd–F distance is too long to produce a well-resolved ^19^F ENDOR doublet when measured at the central transition. By focusing on the |−5/2⟩ ↔ |−3/2⟩ and |−7/2⟩ ↔ |−5/2⟩ EPR transitions, a resolution enhancement of 4.5- and 7-fold was obtained, respectively. We also present data analysis strategies to handle contributions of different electron spin manifolds to the ENDOR spectrum. Our new extended ^19^F ENDOR approach may be applicable to Gd–F distances as large as 20 Å, widening the current ENDOR distance window.

## Introduction

Pulse electron paramagnetic resonance (EPR) methods, particularly those relying on electron–electron dipolar interactions (pulse dipolar EPR, PD-EPR) have emerged as effective tools for providing structural information on proteins and nucleic acids.^1,2^ PD-EPR experiments, usually carried out on frozen solutions, yield distance distributions between two paramagnetic centers.^3^ Since most biomolecules are diamagnetic, paramagnetic spin labels have to be introduced.^4^ The positions for installing labels are primarily selected based on the biological/structural question under consideration, and the choice of the spin label is dictated by the needs for chemical stability and/or compatibility with the biomolecule in its environment. In the last two decades, the number and variety of spin labels has expanded significantly and, at present, comprise nitroxide spin labels, trityl radicals,^5,6^ and Gd(III),^7^ Cu(II),^8^ and Mn(II)^9^ complexes. For routine experimental setups, the distance range accessible by the PD-EPR methodology is 2–6 nm. The long-range limit can be extended by applying rather complicated pulse sequences, like the 7-pulse double electron–electron resonance (DEER) sequence,^10^ or by deuterating the protein.^11^ The short limit can be extended down to 1.5 nm by applying sufficiently short microwave pulses, preferably with single-resonance techniques^12,13^ or reverting to continuous-wave (CW) EPR, where broadening induced by a pair of labels is isolated via comparison with the width of two singly labeled proteins.^14^ Alternatively, rather than measuring electron–electron dipolar interactions, electron–nuclear dipolar interactions can be targeted by applying electron–nuclear double resonance (ENDOR) approaches. In ENDOR, hyperfine interactions between the electron spin in a paramagnetic center and the surrounding nuclei are measured. Traditionally, it has been applied to extract the local spatial and electronic structure of intrinsic paramagnetic metal ions or metal clusters in proteins,^15−20^ while, at present, ENDOR experiments for distance determination on spin-labeled biomolecules are being developed.^21−27^ For measuring weak hyperfine interactions, which are dipolar in nature, Mims ENDOR^28^ is the technique of choice, as demonstrated for a nitroxide label situated 1 nm away from a ^31^P nucleus in a membrane bilayer.^29^

Bennati and co-workers demonstrated on synthetic models and RNA molecules that distances up to 1.5 nm (15 Å) can be determined by combining nitroxide and ^19^F labeling.^21,30^ The use of ^19^F provides high sensitivity, approaching that of ^1^H, owing to its high gyromagnetic ratio, as well as excellent selectivity, since ^19^F is absent in natural proteins and nucleic acids, in contrast to the abundant ^1^H. This feature was previously harnessed to investigate the binding of fluorinated substrate analogues to intrinsic paramagnetic centers in metalloenzymes by ENDOR.^31,32^ In addition, ^19^F labeling is notably more benign than attaching spin labels and can be performed at any predetermined location in a protein, even in its core. This contrasts with large spin labels that are attached to surface residues, are flexible, and therefore significantly enlarge the sampled distance distribution, lowering resolution, especially for short distances. Carrying out ^19^F ENDOR measurements at the W-band (95 GHz) has the advantage that the separation between ^19^F and ^1^H signals is sufficiently large to avoid the overlap encountered at the Q-band (34 GHz).^33^ ENDOR measurements of nitroxides at the W-band are complicated by the resolved *g*-anisotropy, which leads to orientation selection, i.e., preferential excitation of spin labels with certain orientations at different magnetic field positions within the EPR spectrum. Therefore, distance determination requires acquiring a series of ENDOR spectra at different fields, which is time-consuming, particularly for distances larger than 10 Å. Nevertheless, orientation selectivity permits an accurate determination of the parallel component of the dipolar interaction and thereby provides access to longer distances.^21^ In the case of rigid spin labels, additional structural information can be obtained by determining the orientation of the spin label in the structure of the biomolecule. Trityl^22,25^ and Gd(III)^23,27^ labels have been also used for distance determination by ^19^F ENDOR, with the advantage of not requiring a set of orientation selection measurements owing to the isotropic nature of their EPR spectrum. Recently, phenoxyl^24^ and Cu(II)^26^ have also been used for distance measurements. Going beyond *in vitro* measurements, in-cell Gd(III)–^19^F ENDOR significantly expanded the scope of this technique.^27^

When applied to the same systems, ^19^F ENDOR data are complementary to results obtained by ^19^F paramagnetic relaxation enhancement (PRE) and pseudo contact shift (PCS) solution nuclear magnetic resonance (NMR) techniques.^34−36^ The latter cover similar distance ranges, are carried out at ambient temperatures, and provide average electron–nuclear distances, while ENDOR measurements are performed in the frozen state and report on the conformational distribution. Furthermore, at least for solution NMR, the rotation correlation time of the molecule presents a limitation, excluding studies on very large systems, in contrast to ENDOR and magic angle spinning solid state NMR measurements.

At present, the longest distance determined from an ^19^F ENDOR spectrum featuring a resolved doublet with a splitting of 20 kHz was 15 Å in an RNA molecule with a semirigid nitroxide spin label.^21^ In general, distances extracted from ENDOR measurements are limited by the intrinsic widths of the ^19^F ENDOR lines, which generally range from 20 to 35 kHz.^21−23,27,30,37^ The intrinsic line widths are determined by the transverse relaxation of the nucleus^38^ (estimated to be on the order of tens of microseconds^39^), by the electron spin–lattice relaxation time,^38^ and by the width of the electron–nuclear distance distribution.^21^ The bandwidth of the radiofrequency (RF) pulse in the pulsed ENDOR experiment can lead to additional broadening, and care should be taken to use sufficiently narrow band pulses.^38,40^ Smaller hyperfine couplings (longer distances) with unresolved doublets can, in principle, be estimated by measuring absolute Mims ENDOR efficiency;^23^ however, the reliability of such intensity measurements is yet to be demonstrated.

**Figure 1:**
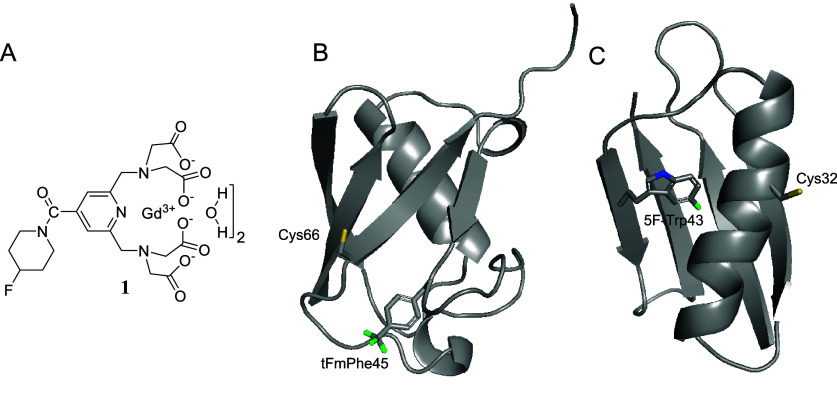
(A) Chemical structure of a molecular ruler for ENDOR Gd–F distance measurements. (B, C) Backbone structures in ribbon representation of ubiquitin T66C (pdb id 1UBQ(42)) (B) and GB1 Q32C (pdb id 1GB1(43)) (C). The cysteine- and fluorine-containing side chains are shown in stick representation with the sulfur atoms colored yellow and the fluorine atoms in green.

Here, we put forward a different approach for extending the long-distance range of ENDOR measurements by increasing the frequency resolution of the ^19^F doublet. We exploit high-spin Gd(III) labels and accessing EPR transitions other than the central transition (CT, |−1/2⟩ ↔ |+1/2⟩),^41^ facilitated by a high magnetic field and low temperatures. We demonstrate the viability of this approach on a specially designed and synthesized model compound **1** that serves as a molecular “ruler”, shown in Figure 1A, along with two model proteins labeled with ^19^F and Gd(III): ubiquitin (Figure 1B), where the standard ^19^F ENDOR doublet is barely resolved, and the B1 domain of immunoglobulin-binding protein G (GB1) (Figure 1C) with an unresolved ^19^F signal. We show that by focusing on the Gd(III) |−7/2⟩ ↔ |−5/2⟩ transition, the spectral resolution is increased by a factor of ∼7, resulting in extending the distance range by a factor of 1.9. We estimate that this may potentially reach a Gd–F distance of 20–25 Å.

## Experimental Details

The synthesis of Gd–F ruler **1** is described in detail in the Supporting Information (SI, Section S1). In brief, the corresponding PyMTA ((pyridine-2,6-diyl)bis(methylenenitrilo)tetrakis acetate) methylcarboxylate precursor was synthesized according to a literature procedure,^44^ hydrolyzed to the carboxylic acid, and amidated with 4-fluoropiperidine. Upon removal of protecting groups, it was complexed with Gd(III), yielding complex **1**. The predicted Gd–F distance for this compound is 9.5–10.0 Å based on quantum chemical calculations (SI, Section S2).

Proteins were prepared and spin-labeled as described previously.^27^ Ubiquitin T66C possesses 4-trifluoromethyl phenylalanine (tFmPhe) at position 45, and GB1 Q32C contains 5-fluorotryptophan (5F-Trp) at position 43. The BrPSPyDO3A-Gd(III) tag was attached to the single cysteines on both proteins.^45^ Chemical structures of the Gd(III) tag and F-labeled amino acids are shown in Figure S1 (SI).

Complex **1** was dissolved in 50:50 v/v D_2_O/glycerol-*d*_8_ solution at a final concentration of 380 μM. Proteins were dissolved in 25 mM D_2_O-based phosphate buffer (pD 7.0) and 150 mM NaCl, and 20 vol % glycerol-*d*_8_ was added as a cryoprotectant, yielding a final protein concentration of 40 μM. For EPR measurements, solutions (ca. 3 μL) were placed in fused silica capillaries (inner diameter 0.6 mm) and sealed at one end.

Pulsed EPR and ENDOR measurements were performed using two pulsed W-band EPR spectrometers, an upgraded home-built EPR/DNP spectrometer,^46^ permitting EPR measurements at 1.7–300 K and ENDOR measurements at 6–300 K, and a Bruker Elexsys E680 spectrometer equipped with a home-built W-band MW extension and a cryogen-free Cryogenic 6 T magnet with a variable-temperature insert that allows ENDOR measurements at 2.2–300 K. Detailed descriptions of ENDOR experimental conditions are provided in the SI (Section S4). The RF pulse length was chosen to prevent significant line broadening caused by the finite RF pulse bandwidth and ensure an acceptable signal-to-noise ratio (SNR). The RF pulse length chosen for experiments with complex **1** (*t*_RF_ = 30 μs) results in a very small broadening of ca. 5 kHz (Figure S2) that does not impair resolution, while for the protein samples no such broadening was observed (see Figure S3 of ref (27)).

As shown earlier,^47^ the use of an adiabatic chirp pulse prior to the Mims sequence allows for the transfer of spin polarization to the observed electron spin transition. For the systems studied here, at low temperatures, this results in a signal enhancement of ca. 30% (Figure S3) at 6 K, but due to instrumental reasons, the use of chirp pulses in the current setup also increases the noise level to a small degree. In addition, this complicates the quantitative determination of the contribution from each EPR transition to the ENDOR spectra. Therefore, all spectra in this work were obtained in the absence of adiabatic chirp pulses.

Details of the numerical simulations of the echo-detected EPR spectra (ED-EPR) and Mims ENDOR spectra are presented in the SI (Section S6), and the best-fit parameters obtained from the simulations are listed in Tables S3 and S4.

## Results and Discussion

### Theoretical Background

**Figure 2:**
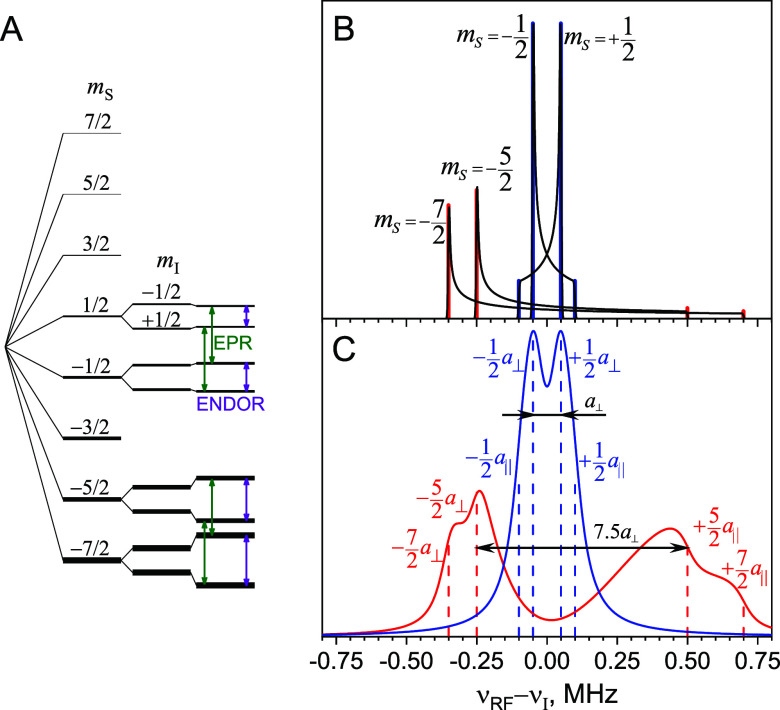
(A) Schematic illustration of energy levels corresponding to different projections *m*_*S*_ and *m*_*I*_ of electron and nuclear spins for Gd(III) coupled to the ^1^H or ^19^F nucleus. Allowed EPR and NMR (ENDOR) transitions are shown with green and violet arrows, respectively. Nuclear sublevels are shown for clarity only for *m*_*S*_ = −7/2, −5/2, −1/2, and +1/2. Note, the splittings are not shown to scale. The populations of the energy levels are represented by different line thickness. (B) Simulated powder ENDOR spectra for |−1/2⟩ ↔ |+1/2⟩ and |−7/2⟩ ↔ |−5/2⟩ transitions; vertical blue and red lines mark parallel and perpendicular contributions for |−1/2⟩ ↔ |+1/2⟩ and |−7/2⟩ ↔ |−5/2⟩, respectively. (C) Simulated Mims ENDOR patterns for the |−1/2⟩ ↔ |+1/2⟩ (blue) and |−7/2⟩ ↔ |−5/2⟩ (red) transitions. The dashed lines mark the positions of the singularities corresponding to parallel and perpendicular orientations for |−1/2⟩ ↔ |+1/2⟩ and |−7/2⟩ ↔ |−5/2⟩ EPR transitions. For all spectra, the following parameters were used: *a*_⊥_ = 100 kHz, τ = 2 μs, and a Lorentzian line width of 50 kHz.

We consider the Mims ENDOR powder line shape for a high-spin paramagnetic *S* = 7/2 center, coupled to a nuclear spin with *I* = 1/2. For Gd(III) with weakly coupled nuclei in a high magnetic field, the Larmor frequency of the electron spin, ν_S_, is much larger than that of the zero field splitting (ZFS) components, and the Larmor frequency of the nuclear spin, ν_I_, is much larger than that of the hyperfine coupling. Accordingly, the projections *m*_*S*_ and *m*_*I*_ of the electron and nuclear spins on the external magnetic field axis are good quantum numbers, and the ENDOR resonance frequencies for allowed NMR transitions (|Δ*m*_*I*_| = 1) are given by^41^
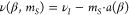
1where β is the angle between the magnetic field and the vector connecting the Gd(III) ion and ^19^F nucleus. Here we consider only long Gd–F distances in nonconjugated systems; hence the hyperfine splitting can be assumed to be purely dipolar, given by
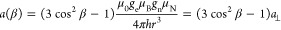
2where μ_0_ is vacuum magnetic permeability, *g*_e_ and *g*_n_ are electron and nuclear *g*-values, μ_B_ and μ_N_ are Bohr magneton and nuclear magneton, respectively, *h* is the Planck constant, and *r* is the Gd–F distance. The energy level diagram for *S* = 1/2 coupled to a nucleus with *I* = 1/2, focusing on the *m*_*S*_ = −7/2, −5/2, −1/2, and +1/2 levels, is shown in Figure 2A.

For each allowed EPR transition, |*m*_*S*_⟩ ↔ |*m*_*S*_ + 1⟩, and each orientation β, the ENDOR spectrum consists of a doublet separated by *a*(β). In a homogeneous frozen solution, all values of β are equally probable and the resulting powder patterns for the EPR transitions |*–*1/2⟩ ↔ |+1/2⟩ and |*–*7/2⟩ ↔ |−5/2⟩ are presented in Figure 2B. In the simulated spectra, the singularities corresponding to parallel (*a*_||_, β = 0) and perpendicular (*a*_⊥_, β = π/2) orientations of the electron–nuclear dipolar vector are highlighted in Figure 2B. Note that the singularities corresponding to *a*_⊥_ and *a*_||_ that belong to the same electron spin manifold *m*_*S*_ appear at different sides of the Larmor frequency, ν_*I*_.

In order to correctly describe the Mims ENDOR spectrum, one has to account for blind spots^40^ and scale each of the ENDOR doublets by the coefficient:
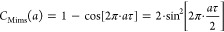
3where τ is the time delay between the first and the second π/2 pulses in the Mims sequence.

The overall Mims ENDOR line shape, *F*_ENDOR_, is obtained by summation over all orientations and EPR transitions:
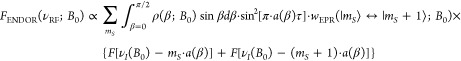
4where *w*_EPR_ is the excitation probability of a given EPR transition and *F* is the line shape of the individual ENDOR line, which, in the general case, can be described by convolution of Gaussian and Lorentzian line shapes.^48^ ρ(β; *B*_0_) is an orientation selection function that represents the number density of Gd–F pairs with orientation β, excited at a particular magnetic field position, *B*_0_, which is uniform (ρ ≡ 1, no orientation selection) in the simplest case.

In Figure 2C we present Mims ENDOR line shapes simulated according to eq 4 for the CT, |−1/2⟩ ↔ |+1/2⟩, and the |−7/2⟩ ↔ |−5/2⟩ transition of Gd(III). For spectra recorded at CT the most pronounced splitting corresponds to *a*_⊥_, given by the difference between the perpendicular singularities of the *m*_*S*_ = ±1/2 manifolds. For other transitions, a different behavior is noted: dominant splitting is observed between parallel and perpendicular features of the spectrum, separated by a strong blind spot in the region of the Larmor frequency. The splitting is on the order of 3|*m*_*S*_^′^|*a*_⊥_, with |*m*_*S*_^′^| = min{|*m*_*S*_|, |*m*_*S*_ + 1|}. Thus, for Gd(III), *S* = 7/2, at a low temperature, where the lowest *m*_*S*_ is predominantly populated, the expected splitting can be as high as 7.5*a*_⊥_ and the longest measurable distance is increased by a factor close to 2 compared to conventional spin 1/2 labels.

Note that one has to be cautious applying the theory of Mims ENDOR blind spots that was developed for species with *S* = 1/2 to high-spin systems. In the case of Gd tags studied here, the validity of this approach is justified, since for most observed spins the value of ZFS is much larger than the bandwidth of the microwave pulses. For this reason, transitions corresponding to different EPR transitions, |*m*_*S*_⟩ ↔ |*m*_*S*_ + 1⟩, can be considered as quasi spin 1/2 systems, with transition probabilities scaled by . Therefore, the Mims ENDOR blind spot behavior in such four-level systems, as illustrated in Figure 2A, is expected to be analogous to that of an *S* = 1/2 system. This was confirmed experimentally earlier for Gd(III) by 2D Mims ENDOR measurements that showed the same modulation frequencies as a function of τ for all transitions.^49^

### ^19^F ENDOR of the Gd–F “Ruler”

**Figure 3:**
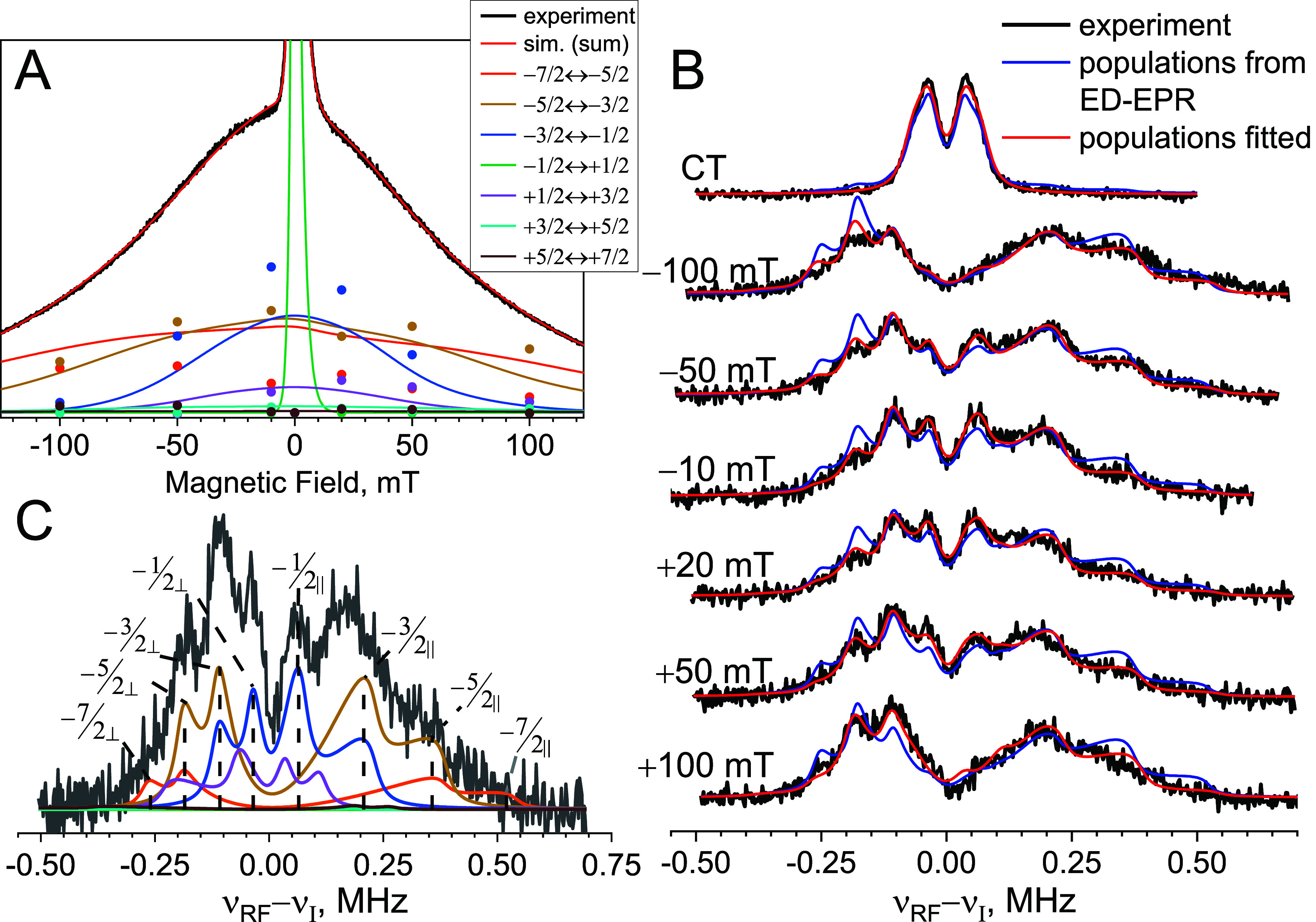
(A) Experimental (black trace) and simulated (red trace) ED-EPR spectra of **1** at 6 K with contributions of individual transitions in different colors. Colored dots correspond to the positions where ENDOR spectra were measured and indicate the transition intensities obtained from simulations of ENDOR spectra shown in panel B. (B) Mims ENDOR spectra (6 K, τ = 2 μs) recorded at different field positions relative to the maximum of the CT (black traces). Simulated spectra using relative contributions of the various transitions determined from simulation of the ED-EPR spectrum (blue traces) were superimposed on simulations with those determined from the ENDOR spectra (red traces). (C) Contributions of individual transitions to the ENDOR spectrum recorded at +50 mT. The color coding for individual transitions is identical in panels A and C, and each of the spectral singularities is indicated by a dotted line in C.

To assess the validity of the proposed approach, complex **1** (Figure 1A) was synthesized, serving as a Gd–F “ruler”. The ED-EPR spectrum of **1** consists of a sharp peak corresponding to the Gd(III) CT, superimposed on a broad envelope that corresponds to all other transitions (Figure 3A and Figure S4A). The contributions of the individual transitions to the ED-EPR Gd(III) spectrum can be deconvoluted by simulations shown in Figures 3A and S4A and parameters listed in Table S3. To ensure that contributions of the individual transitions to the ENDOR spectra can be correctly determined, the ED-EPR was acquired with the Mims ENDOR pulse sequence, the same time delays with the RF frequency set away from the ^1^H and ^19^F resonances (Figure S5). This was necessary because different transitions can have different phase memory times, and consequently ED-EPR spectra acquired with different pulse sequences may have somewhat different line shapes.^50^ Echo decay and spin–lattice relaxation measurements carried out on the central transition are presented in Figure S4.

^19^F Mims ENDOR spectra of **1** recorded at different magnetic field positions are presented in Figure 3B. The top spectrum, recorded at CT, shows a doublet with a resolved splitting of *a*_⊥_. This spectrum is well reproduced by simulations, assuming that it arises only from the CT, yielding *a*_⊥_ = 73.7 ± 0.9 kHz and a Lorentzian line width Δ_L_ = 19.0 ± 0.9 kHz.

The other spectra recorded away from the CT (Figure 3B) exhibit complex shapes with several peaks. We simulated these using eq 4, with the *a*_⊥_ and line width parameters from the simulation of the CT spectrum and the contributions of the individual EPR transitions from the simulation of the ED-EPR spectrum (see colored lines in Figure 3A). The contributions of the individual transitions to the ENDOR spectra are depicted in Figure 3C for the ENDOR spectrum recorded at +50 mT. At this juncture, we emphasize that the agreement between simulation and experiment is remarkable, given that the simulations of ENDOR spectra recorded off the CT were performed without any fitting parameters and that all parameters were taken from independent experiments. Further improvements can be obtained by a global fitting of *a*_⊥_, the line width, and the relative contributions of each EPR transition, and these improved fits are shown in Figure 3B, with best-fit parameters listed in Table S4, yielding *a*_⊥_ = 76.6 ± 1.1 kHz, which corresponds to *r* = 9.9 ± 0.05 Å. This Gd–F distance is in excellent agreement with the distance obtained by DFT optimization, *r* = 10.03 Å for the axial conformer and *r* = 9.51 Å for the equatorial conformer of **1**, the former being slightly more stable (see Section S2, SI).

The best-fit contributions of the various EPR transitions at each field are shown in Figure 3A as dots. Only minor, albeit systematic, differences are observed between the contributions from the simulations of the ED-EPR spectrum and those from global fitting of the ENDOR spectra. In general, the values obtained from the ED-EPR simulation slightly underestimate the contribution of the |−3/2⟩ ↔ |−1/2⟩ transition and overestimate the contribution of the |−7/2⟩ ↔ |−5/2⟩ transition. The observed discrepancies most likely originate from the uncertainties inherent to ED-EPR spectra simulations, in particular pulse nonideality and the uncertainty in the exact form of the ZFS parameter distribution. In this context, we point out that the transition weights obtained from the ENDOR spectra are independent of these shortcomings and can, in principle, be used to experimentally validate and refine the ZFS distribution models.

To further corroborate the above data analysis approach, we also applied it to the ^1^H ENDOR spectra of **1**. ^1^H ENDOR spectra are complex, since multiple types of hydrogens contribute. Therefore, we initially focused on the analysis of the ENDOR spectrum recorded at the CT for different τ values (Figure S6A,B). Simulations of this series yielded a total of six types of hydrogens. Their tentative assignment based on the DFT-optimized structure of **1**, relative numbers of hydrogen atoms for each type, and Gd–H distances are provided in Figure S6C. We also recorded spectra at several field positions away from the CT (Figure S7) and simulated these with fixed *a*_⊥_, line widths, and relative numbers of hydrogens extracted from the CT measurements and using contributions of the various EPR transitions at each field position obtained from ED-EPR simulations. The agreement between the experimental and simulated spectra is remarkably good (Figure S7), and details of the simulation are provided in Section S8 of the SI.

In summary, the experiments carried out on the Gd–F “ruler” **1** demonstrated the validity of our approach in terms of both data collection and the theoretical model used for analyzing the experimental data. We established that the most advantageous conditions for such measurements are far away from the CT and at very low temperatures, i.e., when the EPR spectrum is dominated by the |−7/2⟩ ↔ |−5/2⟩ transition. Under these conditions, the spectral resolution is the largest, and interpretation of the results is straightforward.

Given the excellent results for the model, we proceeded to apply the above detailed methodology to two protein samples with low-resolution ^19^F ENDOR spectra at the CT arising from longer Gd–F distances and possibly broader Gd–F distance distributions.^27^

### Distance Measurements on Proteins

Two model proteins, T66C ubiquitin and Q32C GB1, that possess tFmPhe and 5F-Trp as fluorinated amino acids, respectively, were tagged with the Gd(III)-BrPy-DO3A spin label, referred to as Ub-T66C-DO3A and GB1-Q32C-DO3A, correspondingly (see Figure 1). The chemical structure of the spin label (Figure S1A) is characterized by a relatively short tether that restricts the conformational mobility of the tag and hence limits the distance distribution width.^45^^19^F ENDOR spectra for the same spin-labeled proteins measured at the CT were reported by us previously, and the ^19^F doublet was barely resolved for Ub-T66C-DO3A and not resolved at all for GB1-Q32C-DO3A.^27^ The detailed comparison between the Gd–F distances obtained from ^19^F ENDOR measured on CT, PRE NMR, and *in silico* modeling of the labeled protein structures was reported in our previous work.^27^ Ub-T66C-DO3A possesses three ^19^F nuclei in the trifluoromethyl group, and in the spectral analysis we consider them as identical, which is justified if the determined distance is substantially larger than the distance between the F atoms (ca. 2 Å).^23^

#### Ub-T66C-DO3A

**Figure 4:**
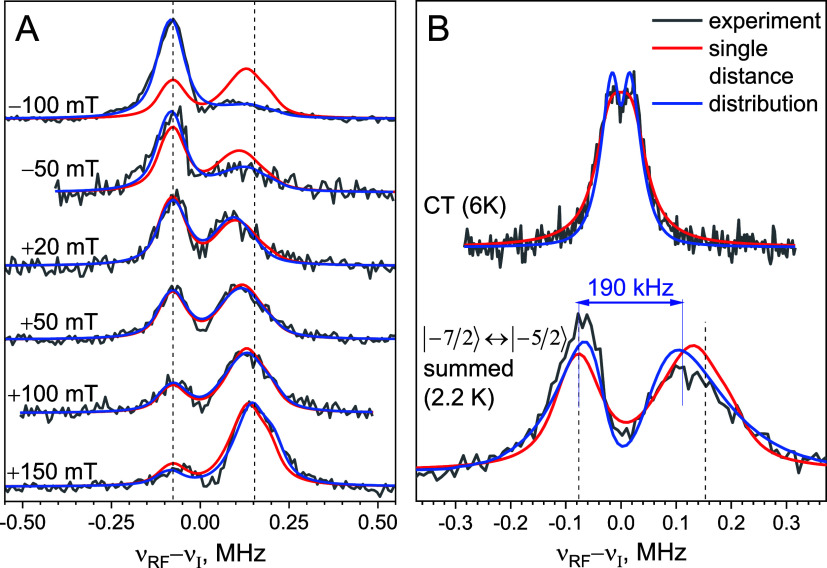
(A) Mims ENDOR spectra of Ub-T66C-DO3A (τ = 4 μs, 2.2 K) recorded at different field positions relative to the maximum of the CT (black trace). Simulated spectra with the orientation selection were taken from simulations of the ED-EPR spectrum (red traces) and represented by the phenomenological function ρ(β; *B*_0_) in eq 4 (blue traces). Note that the experimental spectra recorded at positions −100 and +50 mT CT exhibited a nonuniform background, originating from either remote ^1^H transitions or an instrumental artifact. This background was subtracted in the spectra shown (see Figure S10, SI). (B) Top traces: Mims ENDOR spectrum of Ub-T66C-DO3A (τ = 2 μs, 6 K) recorded at the CT (black trace) and its simulation using a single Gd–F distance (red trace) or a Gaussian distribution of Gd-F distance (blue trace). Bottom traces: summation of all off-CT spectra in (A), weighted according to the ED-EPR spectrum intensity (black trace) along with simulations according to eq 4, with ρ(β; *B*_0_) ≡ 1 and assuming a single Gd–F distance (red trace) or a Gaussian distribution of Gd–F distances (blue trace). Vertical dashed lines in (A) and (B) correspond to parallel and perpendicular singularities of the powder spectra corresponding to *m*_*S*_ = −5/2 electron spin manifold and *a*_⊥_ = 30.6 kHz.

The ED-EPR spectrum and spin relaxation characteristics of Ub-T66C-DO3A are shown in Figure S8. As pointed out above, to enhance resolution, measurements are ideally carried out at the lowest possible temperature. This is clearly illustrated by the temperature dependence of the ED-EPR spectrum of Ub-T66C-DO3A recorded between 1.7 and 16 K (Figure S9A). The associated redistribution of the *m*_*S*_ level populations (Figure S9B) shows that at 2 K the |−7/2⟩ ↔ |−5/2⟩ transition dominates the spectrum, also confirmed by ED-EPR spectrum simulation (Figure S9C,D). Figure 4A shows the Mims ^19^F ENDOR spectra of Ub-T66C-DO3A recorded at 2.2 K at different field positions with respect to the CT. These can be compared to the spectrum recorded at the CT at 6 K (Figure 4B, upper trace), which displays a poorly resolved doublet with a splitting of about 40 kHz. The off-CT spectra exhibit remarkably larger splittings of ca. 190 kHz, with a shape similar to the theoretical predictions illustrated in Figure 2C, and the spectral maxima are identified as 5*a*_⊥_/2 on the left and 5*a*_||_/2 on the right. These are highlighted in Figure 4 by dashed vertical lines (calculated for *a*_⊥_ = 30.6 kHz). The low-frequency maximum of the spectrum is well aligned with the perpendicular singularity, whereas the high-frequency maximum is somewhat shifted for some fields. According to the simulated spectrum in Figure 2C, the observed splitting in the |−7/2⟩ ↔ |−5/2⟩ spectrum corresponds to ca. 7*a*_⊥_ instead of 7.5*a*_⊥_ as anticipated from the parallel and perpendicular singularity positions.

A striking feature of the off-CT spectra is the different relative intensities of the signals at the left and right sides of the Larmor frequency for different field positions, suggesting orientation selection,^29,41,51^ i.e., nonuniform excitation of the differently oriented ZFS over the magnetic field range. This behavior is surprising, considering the broad distribution of ZFS parameters characteristic of Gd(III) complexes.^52^ Indeed, the off-CT ENDOR spectra of complex **1**, which has a different Gd(III) chelate, did not show orientation selection.

The simplest way to analyze the spectra is to abolish the orientation selection by adding all ENDOR spectra with weights equal to the EPR spectrum intensities at the corresponding positions (bottom trace in Figure 4B).^21,53^ The observed splitting of 190 kHz corresponds to *a*_⊥_ ≈ 190/7 = 27 kHz, yielding a Gd–F distance of *r* ≈ 14 Å. If a sufficient number of spectra are summed, the line shape can be analyzed using eq 4 without any orientation selection (ρ ≡ 1). The numerical simulation using eq 4 (Figure 4B) yielded *a*_⊥_ = 30.1 kHz and *r* = 13.5 Å. Using a Gaussian distance distribution with *r*_0_ = 14.9 Å as the center of the distance distribution and Δ*r* = 4.1 Å as its width improves the agreement between the simulation and the experimental spectra (Figure 4B). This distance distribution is in excellent agreement with the one derived from the central transition ENDOR, namely, *r*_0_ = 14.8 Å and Δ*r* = 6.3 Å.^27^

Despite the difference in the average distances obtained by both approaches (i.e., distribution vs single distance), similar spectral shapes are obtained, since shorter distances contribute more to the overall ENDOR spectrum due to the ∼1/*r*^6^ electron–nuclear distance dependence of the ENDOR efficiency.^27^ As a result, wide distance distributions introduce larger uncertainties in the measured distances (in the present case amounting to ca. 1.5 Å with respect to the mean distance). These uncertainties can be reduced by choosing Gd(III) tags with short tethers, which limits the conformational space sampled by the tag.

It is also possible to analyze the set of spectra in Figure 4 individually, which is usually done when the anisotropy is determined by *g*-anisotropy.^21,26^ In this case, information regarding the orientation of the electron–nuclear dipolar vector in the framework of the *g*-tensor is obtained and, in turn, provides additional important geometrical information.^54^ This approach, however, comes with several caveats for cases when the anisotropy is dominated by the Gd(III) ZFS. First, the predicted orientation selection behavior is sensitive to the details of the ZFS distribution, which can be obtained only to a limited extent from the ED-EPR spectra. Second, the orientation of the ZFS tensor within the Gd(III) moiety is likely to be distributed, resulting in a wide orientational distribution of the Gd–F vectors in the framework of the ZFS tensor of Gd(III).^52^ Adding distributions to the orientations of the dipolar direction relative to the ZFS is impractical, as it should be correlated with the distribution of the ZFS parameters. Nevertheless, we attempted using this approach, assuming a well-defined orientation of the Gd–F vector, with polar angles (θ_F_, φ_F_) in the reference frame of ZFS as fitting parameters (red traces in Figure 4A). This resulted in *a*_⊥_ = 30.4 kHz (θ_F_ = 86°, φ_F_ = 90°), but agreement for −50 and −100 mT was unsatisfactory (see Section S10, SI, for details). Therefore, we preferred to account for the orientation selection by considering directly the orientation of the Gd–F vector relative to *B*_0_. For this, we defined a function ρ(β; *B*_0_) in eq 4, which takes into account the relative number of molecules with an orientation β between *B*_0_ and the Gd–F vector. The orientation selection function ρ(β) for each spectrum was fitted individually, and details of this fitting procedure are provided in the SI (Section S10). Although this approach comprises an increased number of fitting parameters for each spectrum, these parameters are independent, and information on the Gd–F distances can be extracted, as long as the hyperfine splitting and line widths are assumed to be the same for all simulated spectra. The result is shown as blue traces in Figure 4A, revealing excellent agreement between the experiment and the simulation for *a*_⊥_ = 31.4 kHz. The resulting orientation selection functions ρ(β) for different field positions are depicted in Figure S13, revealing that for spectra recorded left of the CT there is preferential excitation for Gd–F vectors perpendicular to the static magnetic field (β = 90°), while to the right of the CT parallel orientations (β = 0) are preferentially excited.

Table 1 lists all *a*_⊥_values obtained from the different analysis approaches, with an average of *a*_⊥_ ≈ 30.6 ± 2.4 kHz, corresponding to an average Gd–F distance of *r* = 13.4 ± 0.4 Å. The small uncertainty in the Gd–F distance suggests that the proposed technique can be exploited to extract distances with high precision. Note that the ENDOR line widths obtained for Ub-T66C-DO3A are somewhat larger than those for the Gd–F ruler (complex **1**) and for GB1-Q32C-DO3A (see Table S4, SI). A possible reason for this may be contributions from three distinct fluorine atoms in tFmPhe, as well as a broader Gd–F distance distribution, as reported earlier.^27^

**Table 1:** Comparison of the *a*_**⊥**_ Values and the Associated Distances of Ub-T66C-DO3A, Determined from Different Analysis Approaches of the ENDOR Spectra Shown in Figure 4, and the Lorentzian Line Widths Δ_L_ Used in the Simulations (Gaussian Line Widths Were Found to Be Δ_G_ = 0 in All Cases)

method	*a*_⊥_, kHz	*r*, Å	Δ_L_, kHz
splitting determination	27	14	
fit of summed spectra; single Gd–F distance^a^	30.1 ± 1.4	13.5 ± 0.2	42 ± 8
fit of summed spectra; Gaussian distribution of distances^b^		*r*_0_ = 14.9 ± 0.5	13 ± 6
		Δ*r* = 4.2 ± 0.6	
fit of individual spectra orientation selection predicted from ED-EPR simulation^c^	30.4 ± 1.6	13.5 ± 0.2	36 ± 5
fit of individual spectra; orientation selection modeled phenomenologically^d^	31.4 ± 2.0	13.3 ± 0.3	38 ± 3

a Figure 4B, red line.

b Figure 4B, blue line.

c Figure 4A, red line.

d Figure 4A, blue line.

For comparison, we also recorded ^19^F ENDOR spectra at 6 K, showing that spectra recorded at positions ±50 mT away from the CT are similar to those recorded at 2.2 K (Figure S14). Therefore, we posit that also at 6 K the contributions of low-lying electron spin manifolds to the ENDOR spectrum are significant and that Gd–F distances can be estimated from such higher temperature spectra. This makes our approach more broadly applicable since low temperatures around 2 K are not readily accessible in many spectrometers.

#### GB1-Q32C-DO3A

**Figure 5:**
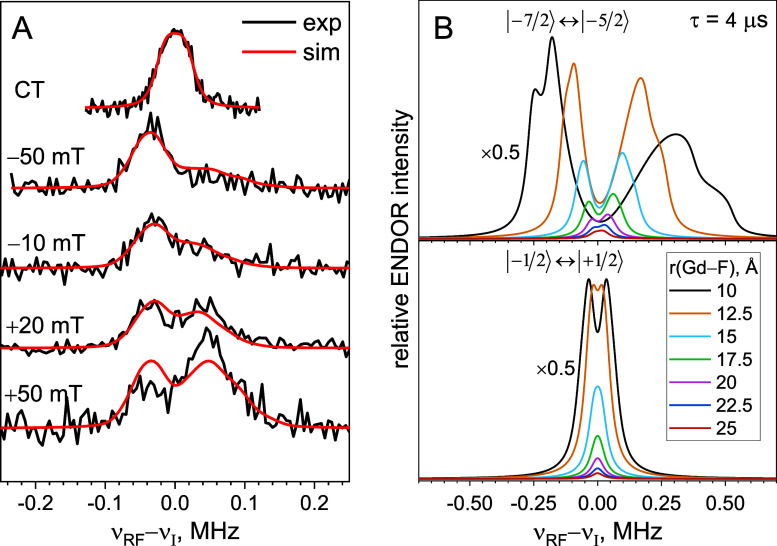
(A) Experimental (black traces) and simulated (red traces) Mims ^19^F ENDOR spectra of GB1-Q32C-DO3A (τ = 4 μs, 6 K) at different field positions with respect to the maximum of the CT. (B) Simulated ^19^F ENDOR spectra illustrating the shape dependence of the spectra for the |−7/2⟩ ↔ |−5/2⟩ (upper panel) and |−1/2⟩ ↔ |+1/2⟩ (lower panel) EPR transitions on the Gd–F distance with Δ_L_ = 30 kHz. The intensity of the spectrum at the shortest distance is scaled by 0.5.

The ^19^F ENDOR spectra of GB1-Q32C-DO3A recorded at 6 K are shown in Figure 5A, and they exhibit a well-resolved splitting of ca. 85 kHz for off-CT, in contrast to the unresolved CT spectrum. The ED-EPR spectrum and relaxation measurements on this protein are presented in Figure S15.

Because of the higher temperature, the contributions of the various transitions have to be taken into account in the analysis of the spectra. It is possible to estimate these contributions from the ED-EPR, although, as shown above for complex **1**, the Gd–F “ruler”, this approach may introduce inaccuracies. Fortuitously, the contributions of different spin manifolds can be readily estimated from an independent experiment, the ^1^H ENDOR spectrum of the same molecule at the same field positions which exhibit a much better SNR. Since the ^1^H and ^19^F ENDOR spectra are recorded with identical parameters (except for the τ values and the RF frequency range), a reliable estimate of the relative contributions for different transitions can be obtained. No orientation selection is expected for the ^1^H ENDOR because of the symmetrical arrangement of the protons around the Gd(III) in DO3A. Another advantage of using ^1^H ENDOR spectra to estimate the EPR transition probabilities is that the protons, being part of the Gd(III) chelate, are not sensitive to the conformation distribution of the spin label within the protein.

In the simulation, two types of hydrogens as well as the matrix hydrogens were considered, consistent with previous ENDOR measurements for Gd complexes^55^ (Figure S16, SI). Hyperfine splittings of individual hydrogens and their relative contributions were determined independently from the spectrum recorded at the CT. The ^1^H ENDOR spectra recorded off CT were simulated to extract the relative contributions of the EPR transitions at different fields (Figure S17), and these values were used in the simulation of the ^19^F ENDOR spectra shown in Figure 5A. Parameters for the simulations are provided in Table S6 along with those obtained from the ED-EPR simulations. As can be appreciated, the ENDOR spectra recorded farthest away from the CT feature orientation selection are similar to those of Ub-T66C-DO3A (compare Figures 5A and S14). Therefore, we assumed that the orientation selection function for GB1 is equal to that previously determined for Ub-T66C-DO3A and is the same for all EPR transitions. Despite such an oversimplification, satisfactory simulations of the ^19^F ENDOR spectra were obtained with the following best-fit parameter: *a*_⊥_ = 21.1 kHz, which corresponds to a Gd–F distance of 15.2 Å. Note that in these ^19^F ENDOR spectra the observed splitting originates mainly from the |−5/2⟩ ↔ |−3/2⟩ electron spin manifold (as illustrated in Figure S18) and, thus, should be on the order of (*a*_⊥_ + *a*_||_)·3/2 = 4.5*a*_⊥_. Therefore, the measured 85 kHz splitting yields *a*_⊥_ ≈ 19 kHz, corresponding to a Gd–F distance of *r* = 15.7 Å, in excellent agreement with the value obtained from the simulation.

#### Estimation of Distance Limits of the Proposed Approach

To evaluate the limits of the above approach, we simulated ^19^F ENDOR spectra for a series of Gd–F distances in the range 10–25 Å with a line width of 30 kHz (Figure 5B) characteristic of the experimental spectra reported here. The upper panel shows spectra recorded at |−7/2⟩ ↔ |−5/2⟩, and the lower panel shows CT spectra (for a *S* = 1/2 system). The CT spectra are resolved for distances up to ca. 12.5 Å, and for the |−7/2⟩ ↔ |−5/2⟩ transition, resolved doublets may be detected up to ca. 20 Å. For a narrower ENDOR line width, ∼20 kHz, the upper distance limit for the CT is expected to be even higher, 15 Å, as is observed for a semirigid nitroxide spin label in RNA.^21^ In this case distances as large as 25 Å may be assessable for off-CT excitation (Figure S19).

As is generally known, the integral Mims ENDOR efficiency scales according to ∼1/*r*^6^, reducing the sensitivity for long *r* values (see Figure 5B). As can be appreciated from Figure 5B, the peak intensity of the ENDOR signal decreases ca. 7-fold, as the Gd–F distance increases from 12.5 to 20 Å. Given the values of SNR per square root time of 4–8 SNR/hour^0.5^, as shown here for Ub-T66C-DO3A at 2.2K (Table S2), one can estimate that for a protein with a Gd–F distance of 20 Å, an SNR of ca. 7 can be obtained within 48 h of acquisition time, making such measurements feasible.

Our approach also comes at the price of lower sensitivity, given the larger width of the EPR spectrum of |*m*_S_| > 1/2 transitions. Higher sensitivity is expected when using rigid Gd(III) tags with small ZFS.^56,57^ In general, given the SNR considerations (Table S2), measurements should be carried out on the CT as long as the ^19^F doublet is resolved, switching to off-CT measurements for cases of unresolved doublets. In addition, improvements in RF efficiency and an increase in the repetition rate, currently limited by the RF amplifier duty cycle, can also be exploited for gaining sensitivity.

Finally, performing these measurements at the Q-band, taking advantage of currently easily accessible pulsed EPR spectrometers, is possible, and ^19^F ENDOR distance measurements at the Q-band have already been reported for trityl- and Cu(II)-labeled biomolecules.^25,26^ However, at lower frequencies, interference between ^19^F and ^1^H ENDOR lines may occur, especially for high-spin electron transitions, since the corresponding ^1^H ENDOR lines can extend far out from the Larmor frequency according to eq 1. If this is the case, subtraction of spectra obtained in the absence of ^19^F is necessary, as recently shown for Cu(II)–^19^F ENDOR.^26^ In addition, the smaller thermal spin polarization at the Q-band may be limiting. To achieve the same thermal occupancy of the low-lying levels at the Q-band, an ∼3 times lower temperature has to be employed compared to the W-band. Since we showed above for GB1-Q32C-DO3A that measurements at 6 K are possible, we envisage that collecting spectra at ∼2 K at the Q-band or by enhancing the population of low-lying spin levels by polarization transfer^47^ is potentially feasible.

## Conclusions

We have developed and presented an efficient approach for significantly extending the range of accessible ENDOR-derived Gd–F distances by taking advantage of the high spin of Gd(III) in combination with high-field and low-temperature measurements. Our approach includes measurement schemes as well as data analysis strategies, as illustrated for a model compound with a fixed Gd–F distance that serves as a molecular “ruler”, as well as for two model proteins containing fluorine atoms and Gd(III) tags. We demonstrate that a Gd–F distance of 15 Å can be extracted from resolved ^19^F ENDOR spectra recorded at the |−7/2⟩ ↔ |−5/2⟩ and |−5/2⟩ ↔ |−3/2⟩ transitions and that distances of up to 20–25 Å may be reachable.

Although a quantitative interpretation of such ^19^F ENDOR spectra in the context of Gd–F distance determination is complex and influenced by orientation selection and overlapping contributions from different electron spin manifolds of Gd(III), we presented effective strategies for overcoming the resulting shortcomings. The contributions of different electron spin manifolds to the ENDOR spectrum can be determined either by EPR spectral simulations or from the ^1^H ENDOR spectrum recorded on the same system. The presence of unexpected orientation selection for the BrPy-DO3A-Gd(III) label in both ubiquitin and GB1 was efficiently treated phenomenologically and did not present an insurmountable problem.

## Data Availability

Source codes and compiled versions of the software used in the present work, as well as the simulation files, are available for download at http://mimsgd.sourceforge.net/.

## Supporting Information Available

Further details on synthesis, EPR and ENDOR measurements, spin relaxation measurements of the samples, spectral simulations, and DFT calculations (PDF)
