# Supplementary material for: Extending the Range of Distances Accessible by 19F Electron–Nuclear Double Resonance in Proteins Using High-Spin Gd(III) Labels
Source: J Am Chem Soc. 2024 Feb 23;146(9):6157–67. doi: 10.1021/jacs.3c13745 (PMC10921402; doi:10.1021/jacs.3c13745)
Supplement: Supplementary file 1 — ja3c13745_si_001.pdf [file ja3c13745_si_001.pdf]

Supporting Information

Extending the range of distances accessible by ^{19}F electron-nuclear double resonance in proteins using high-spin Gd(III) labels.

Alexey Bogdanov[1], Veronica Frydman[2], Manas Seal[1], Leonid Rapatskiy[3], Alexander Schnegg[3], Wenkai Zhu[4], Mark Iron[2], Angela M. Gronenborn[4], Daniella Goldfarb[1*].*

[1] Department of Chemical and Biological Physics, The Weizmann Institute of Science, P. O. Box 26, Rehovot, 7610001, Israel

[2] Department of Chemical Research Support, The Weizmann Institute of Science, P. O. Box 26, Rehovot, 7610001, Israel

[3] Max Planck Institute for Chemical Energy Conversion, 34-36 Stiftstraße, Mülheim an der Ruhr, 45470, Germany

[4] Department of Structural Biology, University of Pittsburgh, 4200 Fifth Ave, Pittsburgh, PA 15260, United States

*E-mail: daniella.goldfarb@weizmann.ac.il, alexey.bogdanov@weizmann.ac.il

Contents

S1. Synthetic details S2
S2. Quantum chemical simulations of complex 1 ..... S5
S3. Structures of Gd(III) and F bearing amino acids S6
S4. Pulsed EPR and ENDOR measurements S7
S5. Polarization transfer using chirp pulses S11
S6. Simulation of EPR and ENDOR spectra S12
S7. Supplementary data on complex 1 ..... S15
S8. Simulation of ^{1}H ENDOR spectra of complex 1 ..... S17
S9. Supplementary data on Ub-T66C-DO3A S20
S10. Orientation selectivity in ^{19}F ENDOR of Ub-T66C-DO3A S23
S11. ^{19}F ENDOR spectra of Ub-T66C-DO3A recorded at 2.2 K and 6 K S27
S12. Supplementary data on GB1-Q32C-DO3A S28
S13. Simulated ENDOR spectra for various Gd–F distances S32
Supplementary References S33

S1. Synthetic details

General Procedures

All reactions were carried out under a nitrogen atmosphere. Column chromatography was carried out on neutral alumina deactivated to the desired degree by addition of the required amount of water to neutral alumina grade I. ^{1}H NMR spectra were recorded on a Bruker Avance 400 spectrometer and chemical shifts are given in ppm referenced to the solvent peak. Mass spectra were recorded as ESI spectra.

Synthesis of complex **1** was performed according to a synthetic route illustrated in Scheme S1.

Scheme S1.

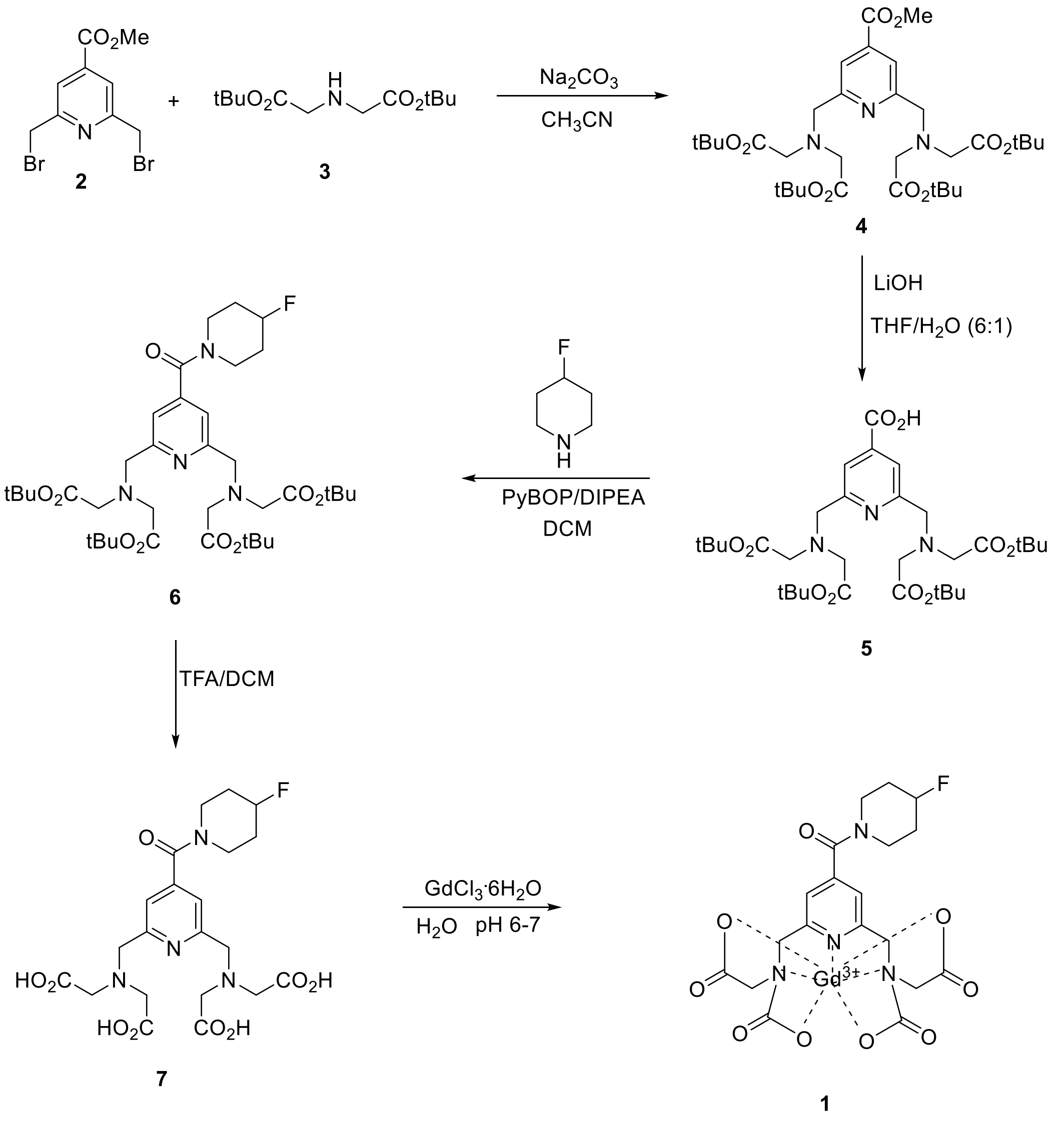

Tetra(*tert*-butyl)2,2′,2″,2‴[4-(methoxycarbonyl)pyridine-2,6-diyl]bis(methylenenitrilo)-tetrakisacetate (4)

Compound **4** was prepared from 2,6-bis(bromomethyl)pyridine-4-carboxylic acid methyl ester (**2**) and di-*tert*-butyl-iminodiacetate (**3**) following the procedure described in the literature.[1] ^{1}H NMR (400 MHz, $CDCl_3$) δ 1.48 (s, 36H), 3.51 (s, 8H), 3.94 (s, 3H), 4.11 (s, 4H), 8.03 (s, 2H).

Tetra(*tert*-butyl)2,2′,2″,2‴-[(4-carboxy)pyridine-2,6-diyl)bis(methylenenitrilo)]-tetrakis acetate (5)

The pentaester **4** (0.862 g, 1.32 mmol) was dissolved in THF (8.4 mL) and a solution of LiOH (35 mg, 1.45 mmol) in H_2O (1.4 mL) was added. The mixture was stirred at room temperature overnight. The THF was evaporated at reduced pressure, the residue was diluted with H_2O (8 mL) and the pH was adjusted to 4.5 by addition of a 1M HCl solution under cooling in an ice-water bath,. The mixture was extracted with DCM and the organic extracts were washed with brine and dried over Na_2SO_4. Evaporation of the solvent under reduced pressure afforded compound **5** as a colorless oil (0.75 g, 88%). ^{1}H NMR (400 MHz, $CDCl_3$) δ 1.48 (s, 36H), 3.53 (s, 8H), 4.13 (s, 4H), 8.11 (s, 2H).

Tetra(*tert*-butyl)2,2',2'',2'''-[(4-(4-fluoropiperidine-1-carbonyl)pyridine-2,6-diyl)bis(methylenenitrilo)]-tetrakis acetate (6)

The acid **5** (0.74 g, 1.16 mmol) was dissolved in anhydrous DCM (10 mL) under nitrogen. To this solution DIPEA (0.75 g, 5.8 mmol) was added, followed by PyBOP (0.97 g, 1.86 mmol). The mixture was stirred for 5 minutes and 4-fluoropiperidine (0.16 g, 1.5 mmol) was added. The mixture was stirred at room temperature under nitrogen overnight and diluted with DCM (30 mL), washed with water (2 x 10 mL), followed by brine (1 x 10 mL) and dried over Na_2SO_4. The solvent was evaporated and the residue was purified by column chromatography on neutral alumina grade II using DCM as eluent. Compound 5 was obtained as a colorless oil (0.41 g, 49%). ^{1}H NMR (400 MHz, $CDCl_3$) δ 1.47 (s, 36H), 1.60-2.0 (br m, 4H) 3.49 (m, 11H), 4.05 (m, 6H), 7.56 (s, 2H).

2,2',2'',2'''-[(4-(4-fluoropiperidine-1-carbonyl)pyridine-2,6-diyl)bis(methylenenitrilo)]-tetrakis acetic acid (7)

The tetraester **5** (0.384 g, 0.53 mmol) was dissolved in anhydrous DCM (12 mL) and TFA (5 mL) was added. The mixture was stirred overnight at room temperature. The solvents were evaporated at reduced pressure and the residue was resuspended in anhydrous DCM (10 mL) and evaporated to remove the remaining TFA (three times). The solid residue was washed with anhydrous ethyl ether followed by anhydrous DCM and dried under vacuum affording the tetraacid **7** in quantitative yield. ^1H NMR (400 MHz, DMSO-d_6) δ 1.50-2.0 (m, 4H), 3.23 (m, 1H), 3.38 (m, 1H), 3.63 (m, 11H), 4.15 (s, 4H), 7.57 (s, 2H).

Gd(III) chelate (8)

The tetraacid **7** (0.1 g, 0.2 mmol) was dissolved in H_2O (3 mL) and $GdCl_3 \cdot 6H_2O$ (82 mg, 0.22 mmol) was added. The pH was adjusted to 6.5 with 0.5M NaOH. The mixture was stirred at room temperature and the pH was maintained between 6 – 7 by addition of small aliquots of 0.1M NaOH. Once the pH remained stable in that range, stirring was continued overnight at room temperature. The precipitate was collected by centrifugation (10,000 rpm, 1 min, 4 °C). The pellet was washed with water (1 mL) and collected by centrifugation at the same conditions as before. The pellet was dried under vacuum affording the chelate **8** as a white solid (30 mg, 23%). MS (ESI^-): m/z 652.07 ($[M-H]^-$), Elemental Composition Analysis (isotope fit): $C_{21}H_{23}FGdN_4O_9$.

S2. Quantum chemical simulations of complex 1

Calculations were carried out using Gaussian16 Rev. C0.1.[2] Geometry optimization was performed at the density functional theory (DFT) level using the Perdew–Burke–Ernzerhof (PBE) functional[3, 4] with the addition of the third version of Grimme and coworkers' empirical dispersion correction[5] using Becke–Johnson dampening (*i.e.*, D3BJ).[6] Density-fitting using Weigend *et al.*'s "universal" Coulomb fitting basis set (known as either W06 or def2/J)[7, 8] was used to increase the computational efficiency of the calculations.[9, 10] The def2-SVP basis set was used with the missing basis set – relativistic effective core potential (RECP) combination for gadolinium[11, 12] taken from the Basis Set Exchange (BSE) library.[13-15] Solvent effects were considered using the SMD implicit solvation model[16] with water as the solvent. The vibrational Hessian matrix was calculated at the equilibrium points to confirm that they correspond to energy minima; a few structures had very small imaginary frequencies ($\lesssim 30i$ cm^{-1}) but the presence frequencies were ignored. Accurate energies were calculated using Zhao and Truhlar's PW6B95 hybrid functional[17] (with incorporates Perdew–Wang's 1991 (PW91) exchange[18-22] and Becke-1995 (B95) correlation[23] functionals) with the fourth version of Grimme's empirical dispersion correction (D4),[24, 25] the SMD solvation model and the def2-TZVP basis set–RECP; D4 corrections were calculated using the code (DFT-D4 version 2.0) provided on Grimme's website.[26]

Previous literature studies[27, 28] show that PyMTA complexes of lanthanides may contain two additional water molecules in the inner coordination sphere. Therefore, two H_2O molecules were explicitly added to the optimized structure to account for the additional coordinated positions. However, during the geometry optimization they consistently moved from the inner coordination sphere to the outer forming hydrogen-bonds with the carboxylic acid groups.

Two geometrical isomers were considered for complex **1**, with the F atom situated in axial and equatorial positions of the piperidine moiety (**Scheme S2**, right and left panels, respectively). Both isomers are very close in energy, with the axial one only ΔG_{298}=0.9 kcal/mol lower than the equatorial one. The predicted Gd–F distances for the two isomers are also very similar, 10.03Å for the axial one and 9.51Å for the equatorial one.

Scheme S2

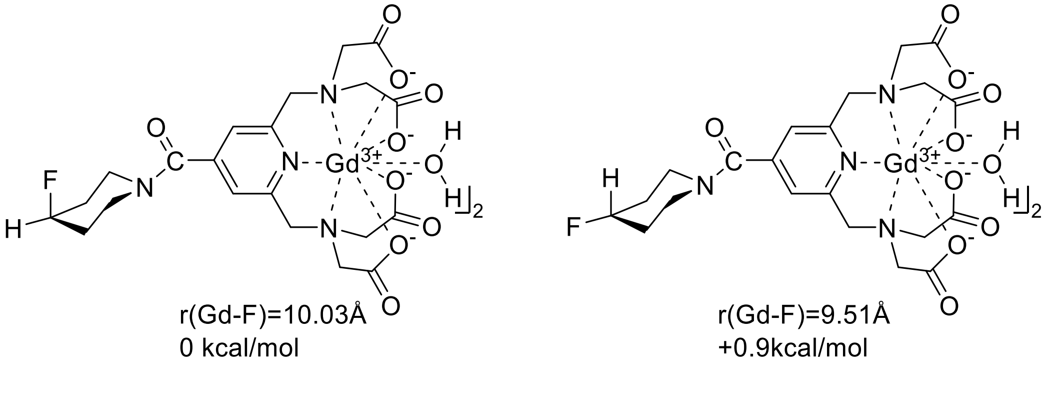

S3. Structures of Gd(III) and F bearing amino acids

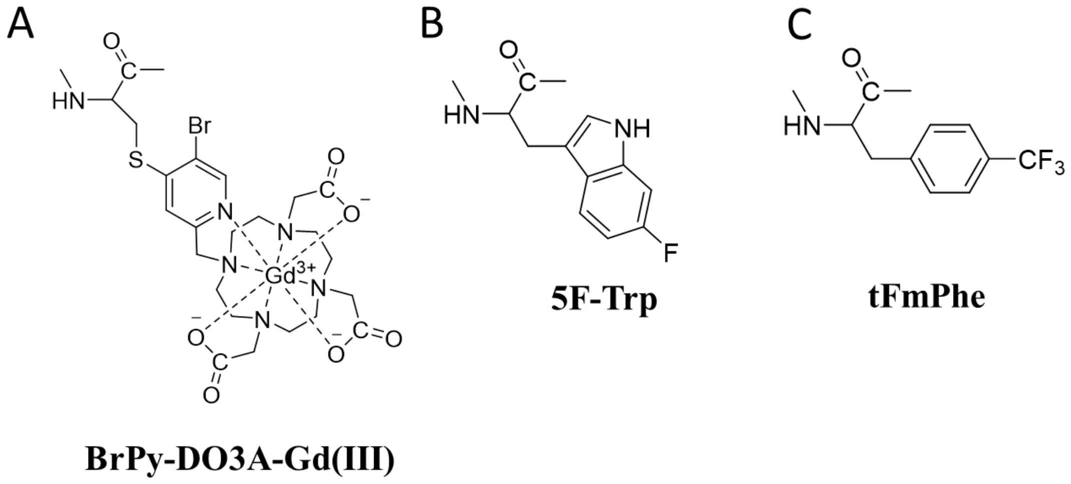

Fig. S1. Chemical structures of amino acid residues corresponding to (A) BrPy-DO3A-Gd(III) labeled cysteine, **(B)** 5-fluoro tryptophan (5F-Trp) and **(C)** p-trifluoromethyl phenylalanine (tFmPhe).

S4. Pulsed EPR and ENDOR measurements

Pulsed EPR and ENDOR measurements were performed using two pulsed W-band EPR spectrometers. The first one (at the Weizmann Institute of Science, WIS) was built on a basis of a 0–5 T cryogen free magnet with integrated variable temperature unit and 300 mT sweep coil (J3678, Cryogenic Ltd.)[29] upgraded to allow for ENDOR measurements using a cylindrical TE_{011} cavity and Helmholtz RF coil as described earlier.[30] It is equipped with 2 W pulsed microwave power amplifier (QPP95023330-ZW1, Quinstar) and a pulsed radiofrequency (RF) amplifier (1 kW, 3446 Herley-AMT, or 2 kW BT02000-GammaS, TOMCO).

ENDOR spectra at 2.2K were recorded using a Bruker Elexsys E680 spectrometer (at Max Planck Institute of Chemical Energy Conversion, MPI) equipped with a home-built W-band bridge employing a 2 W pulsed microwave power amplifier (QPP-94013338MPI, Quinstar), a closed-cycle 6T split pair superconducting magnet with an integrated 2–300 K cryostat (J4233, Cryogenic Ltd.) and a 100 W RF amplifier (ZHL-100W-GAN+, Mini-Circuits).

Echo-detected electron paramagnetic resonance (ED-EPR) spectra were recorded using either Hahn echo ($\pi/2 - \tau - \pi - \tau -$ echo), or stimulated echo ($\pi/2 - \tau - \pi/2 - T - \pi/2 - \tau -$ echo), or Mims ENDOR ($\pi/2 - \tau - \pi/2 - T(\pi_{RF}) - \pi/2 - \tau -$ echo) sequences. For the latter an off-resonance RF pulse was applied.

Mims ENDOR spectra at the WIS spectrometer were recorded at 6 K using the sequence $\pi/2 - \tau - \pi/2 - T(\pi_{RF}) - \pi/2 - \tau -$ echo $- [\tau_2 - \pi - \tau_2 -$ echo$]_n$ with a four-step phase cycle and a Carr-Purcell Meiboom-Gill (CPMG) detection train at the end for enhancing signal-to-noise ratio.[31] Mims ENDOR spectra at the MPI spectrometer were recorded at 2.2 K using the same Mims ENDOR sequence without phase cycling or CPMG detection. In both systems, random sampling of RF was employed,[32] with 10 shots acquired per point. Microwave power was adjusted such as to give a π pulse of 28–32 ns, using Rabi nutation sequence, $t_{nut} - t_{wait} - \pi/2 - \tau - \pi - \tau -$ echo (t_{nut} was varied; t_{wait} was chosen such as to let for the decay of the transverse magnetization). RF power was adjusted to yield a desired π_{RF} pulse length, using a Rabi nutation sequence $\pi/2 - \tau - \pi/2 - T(t_{RF}) - \pi/2 - \tau -$

echo, with a constant mixing time T and varying RF pulse length, t_{RF}. The RF pulse length was set to be long enough to avoid significant broadening of the ENDOR spectrum (**Fig. S2**), while ensuring acceptable SNR. The experimental parameters used for Mims ENDOR acquisition are listed in **Table S1**, and corresponding acquisition times and signal-to-noise ratios for ^{19}F ENDOR are listed in **Table S2**.

Phase memory times of the Gd(III) complexes were estimated by recording the τ dependence of Hahn echo intensity and fitting it to either exponential or stretched exponential function.

Spin-lattice relaxation times T_1 were estimated using the inversion recovery sequence, $\pi - t_{wait} - \pi/2 - \tau - \pi - \tau -$ echo, with varying t_{wait}. The obtained traces were fitted to a stretched exponential function to estimate T_1. Note that this approach leads to an underestimation of the spin-lattice relaxation time due to spectral diffusion.

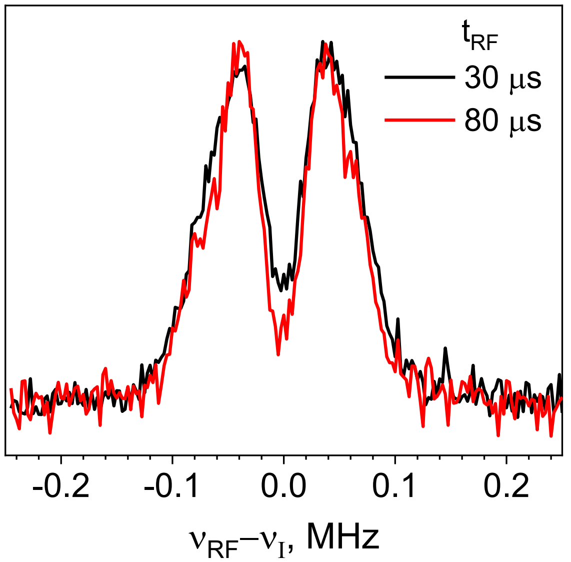

Fig. S2. Mims ^{19}F ENDOR spectra (τ=2 μs) of complex **1** at 8 K recorded with different length of RF pulse t_{RF}.

Table S1. Experimental parameters of Mims ENDOR spectra recording

System	$(\pi/2)_{MW}$, ns	π_{RF}, μs	τ, ns	Shot repetition time, ms
complex **1**, 6K, ^{19}F	14	30	2000	3
complex **1**, 6K, ^{1}H	14	30	120–550	3
Ub-T66C-DO3A, 2.2K, ^{19}F	16	35	4000	10
Ub -T66C-DO3A, 6K, ^{19}F	14	35	2000	2.5
GB1-Q32C-DO3A, 6K, ^{19}F	14	35	4000	3
GB1-Q32C-DO3A, 6K, ^{1}H	14	35	350–550	3

Table S2. Acquisition times and signal-to-noise ratio (SNR)* for ^{19}F ENDOR. A comparison for the different systems is given by SNR per square root of time, as well as by SNR per square root of time multiplied by number of points in the spectrum. Parameters that influence the SNR, such as Gd(III) concentration, number of fluorine atoms per Gd, number of points in the spectrum, are also listed.

system	conc, μM	^{19}F per Gd	SNR	acquisition time, hours	pts in spec-trum	SNR/ (hour)$^{0.5}$	SNR·pts/ (hour)$^{0.5}$
**complex 1, 6K (WIS)**	380	1					
CT –100mT			16.7	13	481	4.6	2230
CT –50mT			16.0	19	481	3.6	1760
CT –10mT			19.3	9	481	6.4	3100
CT			46.3	10.5	401	14.3	5730
CT +20mT			17.3	5	481	7.7	3720
CT +50mT			15.2	6	481	6.2	3000
CT +100mT			19.8	11.5	481	5.8	2800
**Ub-T66C-DO3A, 2.2K (MPI)**	40	3					
CT –100mT			65.4	89	150	6.9	1040
CT –50mT			11.2	6	120	4.6	550
CT +20mT			13.5	6.5	150	5.3	790
CT +50mT			17.8	19	150	4.1	610
CT +100mT			13.5	3	125	7.8	970
CT +150mT			33.6	35	150	5.7	850
**Ub-T66C-DO3A, 6K (WIS)**	40	3					
CT –50mT			16.2	30	241	2.9	710
CT –5mT			16.0	14	241	4.3	1030
CT			20.3	3.5	241	10.9	2620
CT +5mT			20.0	16	241	4.9	1180
CT +50mT			21.4	30	161	3.9	630
**GB1-Q32C-DO3A, 6K (WIS)**	40	1					
CT –50mT			12.7	34	101	2.2	220
CT –10mT			10.0	20	121	2.2	270
CT			16.6	2	101	11.7	1180
CT +20mT			12.2	25.5	121	2.4	240
CT +50mT			9.9	28	161	1.9	300

*The SNR was calculated as the ratio of minimum-to-maximum intensity of the ENDOR spectrum to the mean squared deviation calculated at the background "tails" of the spectrum.

S5. Polarization transfer using chirp pulses

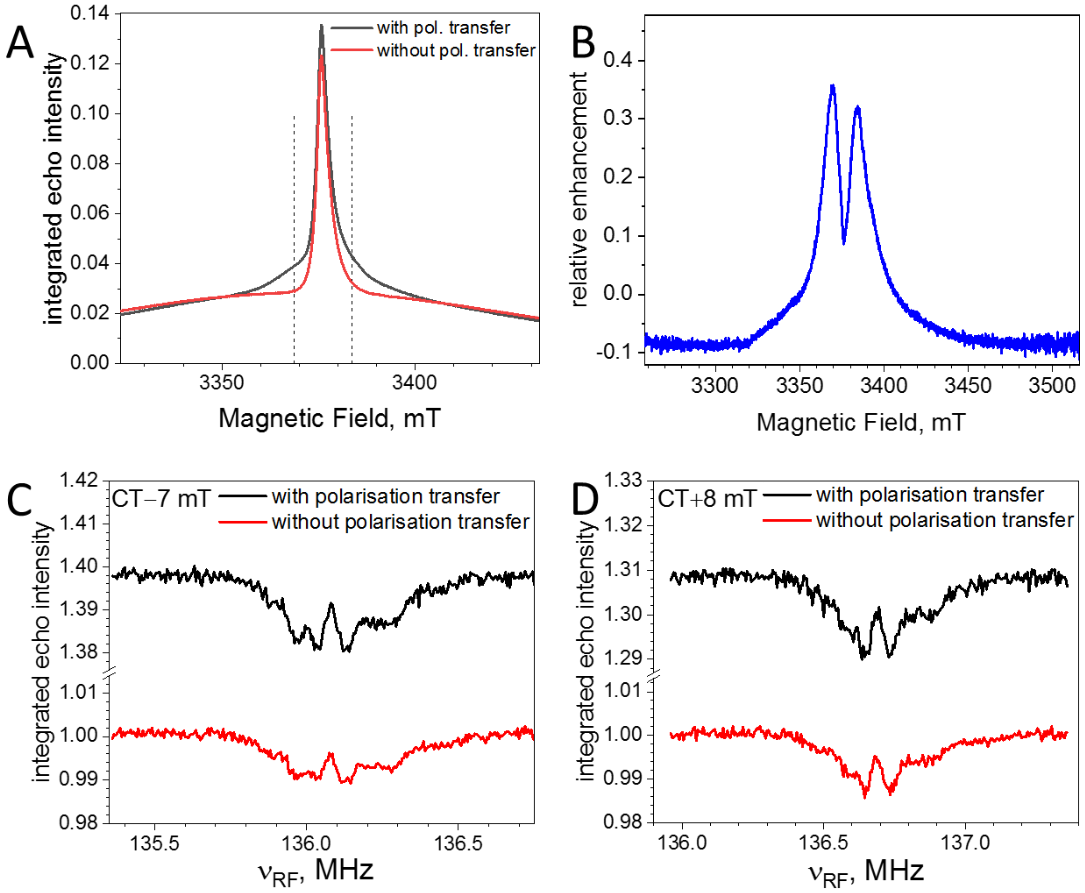

Fig. S3. Polarization transfer in complex **1** using adiabatic chirp pulses. **(A)** Hahn echo detected EPR spectra of **1** in the absence (red trace) and in the presence of two consecutive pre-polarization pulses in the sequence (black trace); pulse parameters were as follows: 1 – chirp 94.70–94.85 GHz, 2 – chirp 94.95–95.10 GHz, each pulse 3 μs length, 5th order WURST; τ=500 ns. **(B)** Magnetic field dependence of the relative echo intensity increase through polarization transfer. **(C,D)** Mims ^{19}F ENDOR spectra of complex **1** in the absence (red traces) and in the presence (black traces) of pre-polarization pulses, at field positions –7 mT **(C)** and +8 mT **(D)** with respect to the CT; pre-polarization pulse parameters same as in panel **A**.

S6. Simulation of EPR and ENDOR spectra

The ED-EPR spectra were simulated using a home written program with the following spin Hamiltonian (in frequency units):

$$\hat{H}_0 = \frac{\mu_B g}{h} \cdot \mathbf{B}_0^T \cdot \hat{\mathbf{S}} + \hat{\mathbf{S}}^T \cdot \mathbf{D} \cdot \hat{\mathbf{S}} \tag{S1}$$

where $\hat{\mathbf{S}}$ is electron spin operator, g is the g-value, $\mathbf{D}$ is the zero field splitting (ZFS) tensor, μ_B is the Bohr magneton and h is the Planck constant. For each orientation of the external magnetic field in the reference frame of ZFS, the eigenvalues of this spin Hamiltonian were computed for the central value of the magnetic field $\mathbf{B}_0$ using the full matrix diagonalization. Following the high-field approximation, resonance frequencies ν_i were converted to resonant fields as $B_i = \frac{B_0 \nu_0}{\nu_i}$, with ν_0 as the spectrometer frequency.

The intensities of the resonances were scaled to compensate for the difference in the effective nutation angles, corresponding to different electron spin transition propabilities. If the pulse tuning is performed at the central transition, this scaling factor is given by[33]

$$C\left(m_S \leftrightarrow m_S + 1\right) = \sin^3\left(\alpha\pi/2\right), \tag{S2}$$

with

$$\alpha = \sqrt{S\left(S+1\right) - m_S\left(m_S+1\right)} \Big/ \left(S + 0.5\right) \tag{S3}$$

and S=7/2 is the spin of Gd(III); this scaling is valid both for Hahn echo and for stimulated echo ED-EPR spectra. In eq. (S3) $\sqrt{S\left(S+1\right) - m_S\left(m_S+1\right)}$ is the probability of the $\left|m_S\right\rangle \leftrightarrow \left|m_S+1\right\rangle$ transition, and $S+0.5$ is the probability of the $\left|-1/2\right\rangle \leftrightarrow \left|+1/2\right\rangle$ transition. Note that this simple treatment is only valid for cases in which the ZFS tensor components are large, compared to the MW field intensity ν_1 and the $\left|m_S\right\rangle \leftrightarrow \left|m_S+1\right\rangle$ transitions are well defined, permitting the observed resonances to be treated as quasi spin ½ transitions with transition probabilities given above. This assumption holds for most observed spins in the Gd(III) spin labels.

The distribution of ZFS parameters was accounted for phenomenologically, as previously described in detail.[34, 35] In essence, two broad Gaussian distributions of the axial ZFS parameter D were assumed, with positive and negative D values. The centers, widths and relative contributions of the two distributions were varied to achieve the best agreement between the experimental and theoretical spectra; the resulting best fit values are listed in **Table S3**. The ZFS parameter E/D, which characterizes of rhombicity of ZFS, was assumed to be distributed between 0 and 1/3 according to the distribution probability

$$P(E/D) = E/D - 2(E/D)^2 \tag{S4}$$

^1H and ^{19}F Mims ENDOR spectra were computed using eq. (4) from the main text. The best fit values of the parameters are listed in **Table S4**.

Both, for calculating EPR and ENDOR spectra, the individual line shape was modeled as Voigtian, i. e. as a convolution of Lorentzian and Gaussian lines, using the formula:

$$F_V(z) = \sqrt{\frac{2}{\pi \cdot \Delta_G^2}} \operatorname{Re}\left[e^{-z^2} \operatorname{erfc}(-iz)\right] \tag{S5}$$

with

$$z = \frac{x\sqrt{2}}{\Delta_G} + i \cdot \frac{\Delta_L}{\Delta_G}\sqrt{\frac{3}{2}} \tag{S6}$$

Δ_L and Δ_G are Lorentzian and Gaussian components of the line width, and x is the off-resonance field (in case of EPR spectra) or frequency (in case of ENDOR spectra) shift. In the case of purely Lorentzian or Gaussian line shapes this equation reduces to

$$F_L(x) = \frac{1}{\pi} \cdot \frac{\Delta_L\sqrt{0.75}}{x^2 + \left(\Delta_L\sqrt{0.75}\right)^2} \tag{S7}$$

and

$$F_G(x) = \sqrt{\frac{2}{\pi \cdot \Delta_G^2}} \cdot \exp\left[-\frac{2x^2}{\Delta_G^2}\right] \tag{S8}$$

respectively. Using the latter formulae in the case of mostly Lorentzian or Gaussian line broadening reduces computational time. Parameter optimization was accomplished using an adaptive nonlinear least-squares algorithm NL2SOL.[36]

Table S3. Values of D and its distribution as determined from the ED-EPR spectral simulations for the systems studied

System	D_1 , MHz	ΔD_1, MHz	rel. contribution	D_2 , MHz	ΔD_2, MHz	rel. contribution
complex **1**	1120	940	62%	–1070	720	38%
Ub-T66C-DO3A	1210	1050	35%	–1130	1080	65%
GB1-Q32C-DO3A	1370	1620	46%	–1220	1050	54%

Table S4. ENDOR spectra simulation parameters for all systems reported in the present work.

System	$a_\perp$, kHz	r, Å	Δ_L, kHz	Δ_G, kHz	Comments
complex **1**, 6K	73.7±0.9 [a]	10.03±0.05 [a]	19.0±0.9 [a]	0	transition probabilities from ED-EPR simulation (blue line, Fig. 3B)
complex **1**, 6K	76.6±1.1	9.90±0.05	11.1±0.9	15.8±1.0	transition probabilities fitted from ENDOR spectra (red line, Fig. 3B)
Ub-T66C-DO3A, 2.2K	30.4±1.6	13.5±0.2	36±5	0	only $\lvert -7/2\rangle \leftrightarrow \lvert -5/2\rangle$ transition; orientation distribution from ED-EPR simulation (red line, Fig. 4A)
Ub-T66C-DO3A, 2.2K	31.4±2.0	13.3±0.3	38±3	0	only $\lvert -7/2\rangle \leftrightarrow \lvert -5/2\rangle$ transition; orientation distribution fitted from ENDOR (blue line, Fig. 4A)
Ub-T66C-DO3A, 2.2K	30.1±1.4	13.5±0.2	42±8	0	only $\lvert -7/2\rangle \leftrightarrow \lvert -5/2\rangle$ transition; summed spectra at different field positions; single distance (red line, Fig. 4B)
Ub-T66C-DO3A, 2.2K	–	$r_0 = 14.9$Å<br>$\Delta r = 4.2$Å	13±6	0	only $\lvert -7/2\rangle \leftrightarrow \lvert -5/2\rangle$ transition; summed spectra at different field positions; Gaussian distribution of distances (blue line, Fig. 4B)
GB1-Q32C-DO3A, 6K	21.1±1.3	15.2±0.3	5±1	19±5	Transition probabilities from ^{1}H ENDOR; orientation selection parameters from ubiquitin simulation (red line, Fig. 5A)

[a] parameters were determined only from the simulation of the ENDOR spectrum recorded at CT

S7. Supplementary data on complex 1

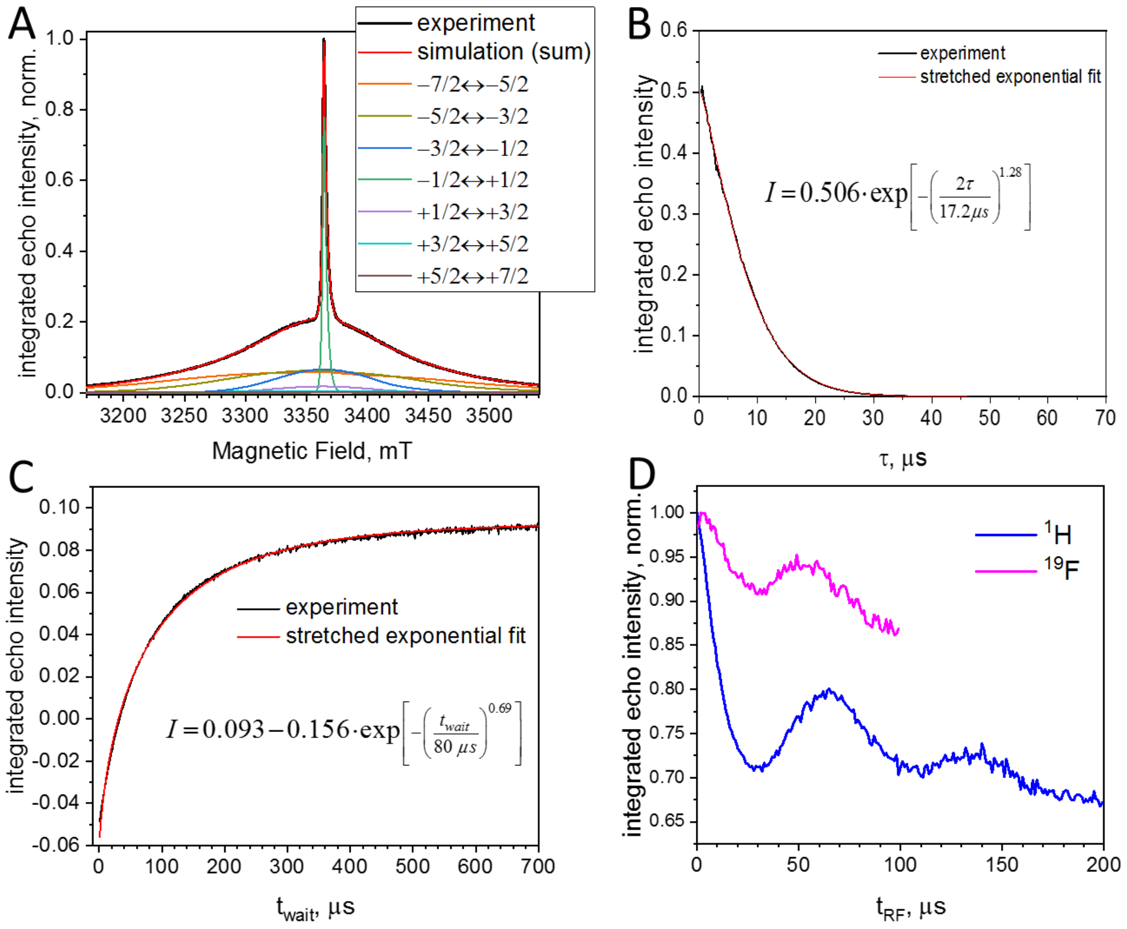

Fig. S4. (A) ED-EPR spectrum of **1** recorded at 6 K (black trace) with simulation (red trace) and the individual contributions of the different EPR transitions (colored lines). The spectrum was recorded using a Mims ENDOR sequence with τ=2 μs, and off-resonance RF ν_{RF} = 128 MHz. The ZFS parameters used in simulation are listed in **Table S3**. **(B)** Echo decay of **1** in 50:50 v/v D_2O/glycerol-d_8 at 6 K measured at CT (black trace) and its stretched exponential fit (red trace). **(C)** Inversion recovery trace of the same sample with a stretched exponential fit (red). **(D)** Rabi nutation measurements using the Mims ENDOR sequence with variable RF pulse length t_{RF}; τ=550 ns, ν_{RF}=144.5 MHz (blue trace) for ^{1}H, τ=4000 ns, ν_{RF}=136.32 MHz (magenta trace) for ^{19}F.

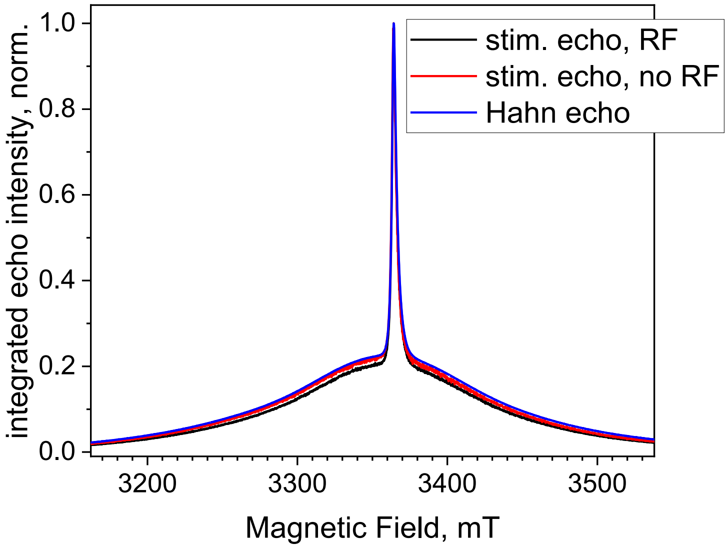

Fig. S5. ED-EPR spectra of **1** (50:50 v/v D_2O/glycerol-d_8, 6 K), recorded using Hahn echo (τ=500 ns; blue trace), stimulated echo (τ=2 μs, T=32 μs; red trace), and Mims ENDOR (τ=2 μs, T=32 μs; ν_{RF}=128 MHz, black trace) pulse sequences. All spectra are normalized with respect to the maximum amplitude.

S8. Simulation of ^{1}H ENDOR spectra of complex 1

Mims ^{1}H ENDOR spectra of **1**, measured at the CT of Gd(III) as a function of the interpulse delay τ, are shown in **Fig. S6A**. These spectra exhibit a complex shape, due to the presence of a number of different protons in the compound. To quantitatively simulate the spectra at least 6 types of inequivalent protons had to be taken into account (**Fig. S6B**). To render the simulation reliable and prevent overfitting, the spectra recorded at 7 values of τ between 120 ns and 550 ns were simulated jointly. The quality of the fit is shown in **Fig. S6A** and the contributions of the individual proton types are illustrated in **Fig. S6B.** The tentative assignment of the proton types is shown in **Fig. S6C**, where the groups of equivalent protons are highlighted on the structure of **1**. The Gd–H distances for each type of proton were estimated from DFT-optimized structure of the complex and are shown next to each proton type in **Fig. S6C**. The values shown are representative of the both geometrical isomers of **1** (with axial and equatorial F in the piperidine moiety). For unambiguous assignments selective deuteration of the PyMTA fragment has to be carried out.

^{1}H ENDOR spectra recorded off CT are shown in **Fig. S7** for two values of τ (350 ns and 550 ns). We simulated these spectra using contributions of the individual EPR transitions, obtained from the simulation of the ED-EPR spectrum (cf. **Fig. 3A**) with all the parameters for individual proton types (hyperfine constant, linewidths, relative contributions) extracted from simulations of the ENDOR spectrum measured at the CT, except for the matrix line (H6 in **Fig. S6B**). The latter was simulated by a Gaussian line centered at the Larmor frequency, with the width and amplitude fitted independently for each spectrum. Such a simple approach resulted in very good agreement between the experimental and theoretical spectra, as can be appreciated from **Fig. S7**. The transition contributions obtained from ^{19}F ENDOR simulation were also tried, and they give the similar quality of fit.

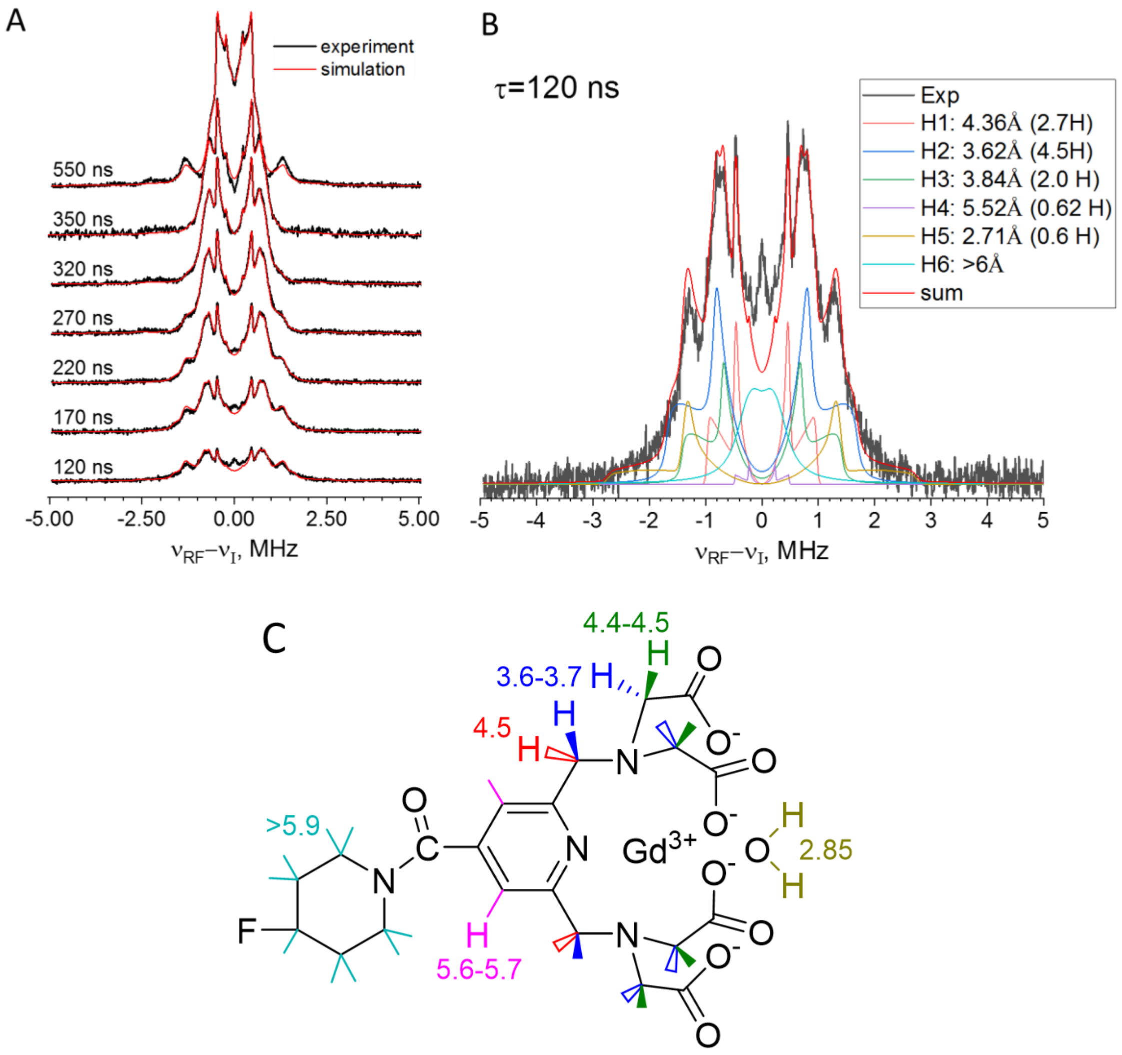

Fig. S6. **(A)** Experimental Mims ^{1}H ENDOR spectra of **1** at different τ values (black traces) and their simulations (red traces). **(B)** Enlarged view of the spectrum recorded with τ=120 ns, with the relative contributions of the 6 proton types, H1–H6; Gd–H distances are listed in the legend, the numbers in parentheses corresponds to the relative amplitude of each proton type. **(C)** Chemical structure of **1** with the different types of protons colored as the traces in panel **B** along with the corresponding Gd–H distances (in Å) obtained from DFT calculations.

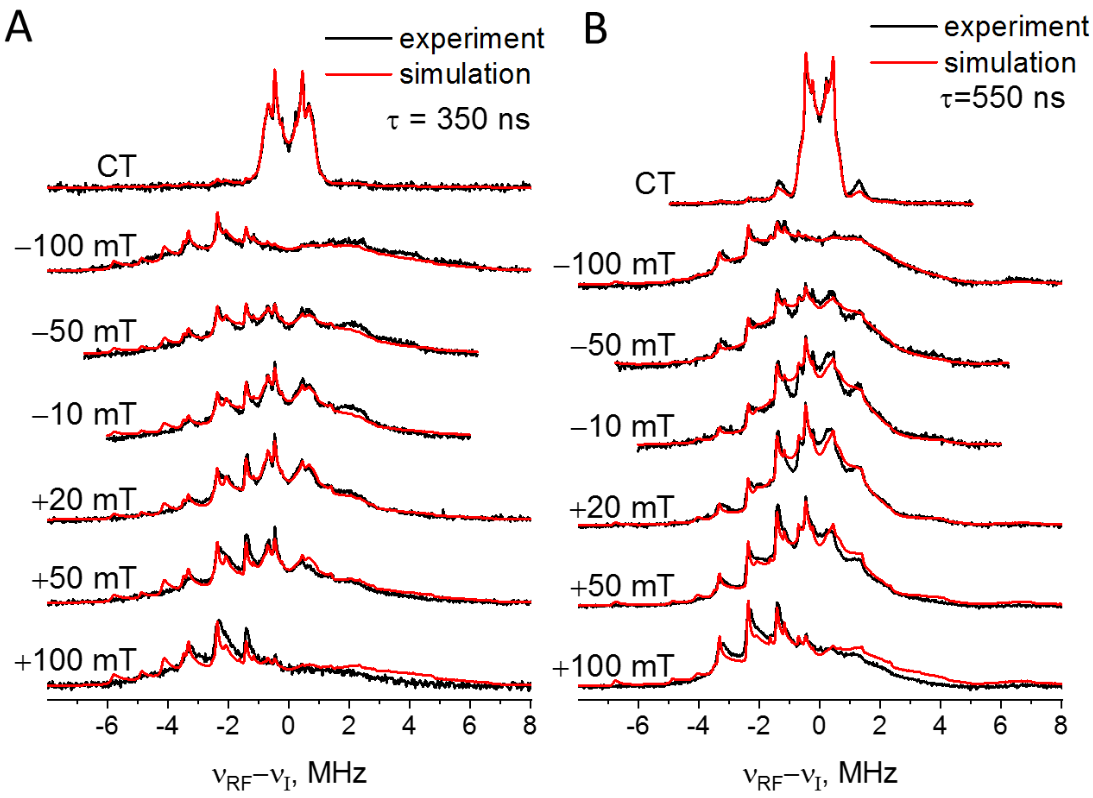

Fig. S7. Experimental Mims ^{1}H ENDOR spectra of **1** (black traces) recorded with τ=350 ns **(A)** and τ=550 ns **(B)** at different magnetic field positions with respect to the CT and their numerical simulations (red traces). The simulations used the proton types, hyperfine splittings and relative contributions from the analysis of the spectra recorded at CT (cf. **Fig. S6**). The relative contributions of the EPR transitions were taken from the simulation of the ED-EPR spectrum (**Fig. S4A)**. No fitting parameters were used.

S9. Supplementary data on Ub-T66C-DO3A

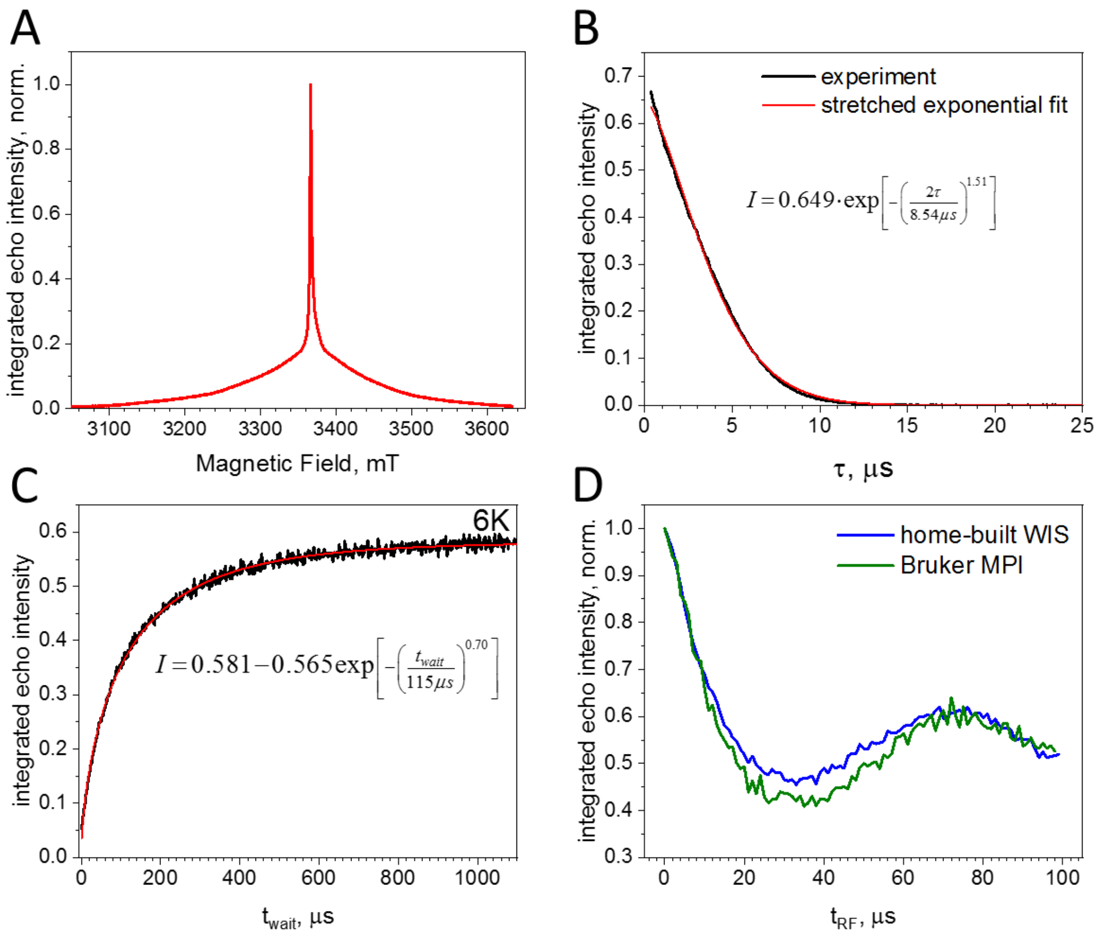

Fig. S8. (A) ED-EPR spectrum of Ub-T66C-DO3A recorded at 6 K. The spectrum was recorded using a Hahn echo sequence with τ=500 ns. **(B)** Echo decay of Ub-T66C-DO3A measured at CT at 6 K (black trace) and its stretched exponential fit (red line). **(C)** Inversion recovery trace of Ub-T66C-DO3A measured at CT at 6 K (black trace) and its stretched exponential fit (red line). **(D)** ^{1}H Rabi nutations, measured at 6 K by Mims ENDOR sequence with variable RF pulse length; τ=550 ns, ν_{RF}=144.5 MHz (WIS spectrometer, blue trace), τ=500 ns, ν_{RF}=143.2 MHz (MPI spectrometer, green trace).

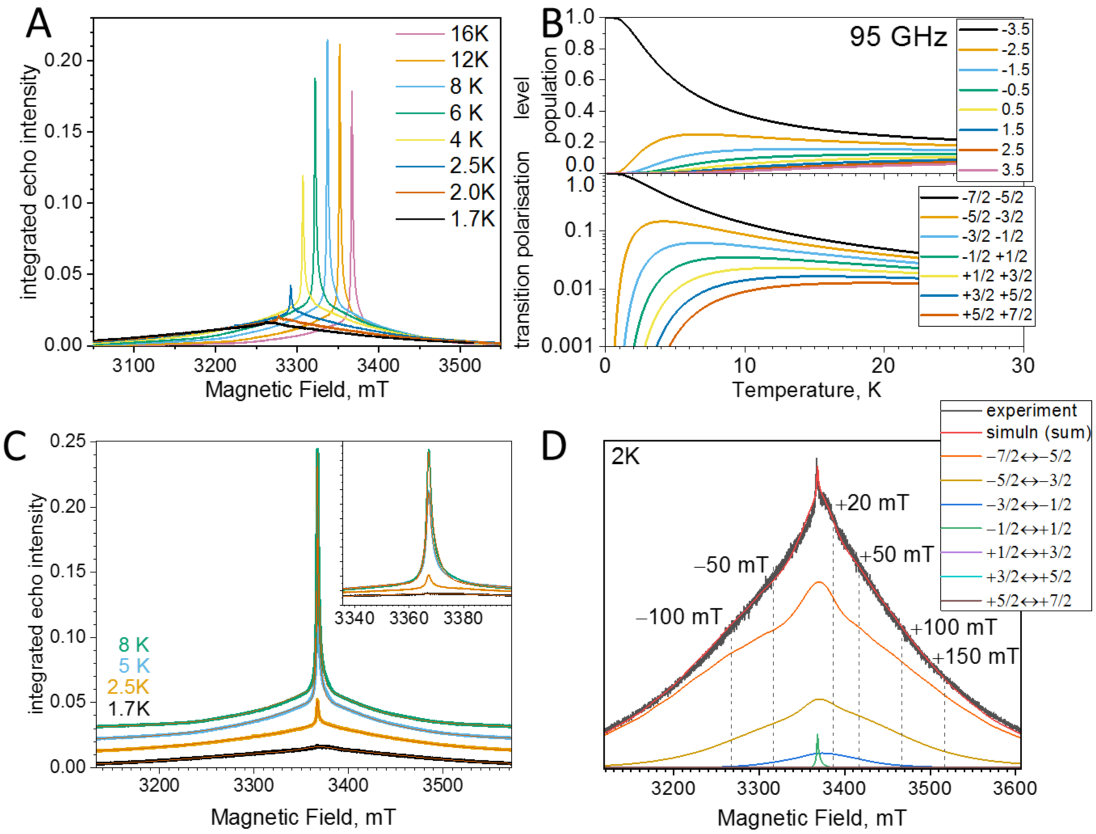

Fig. S9. (A) Experimental ED-EPR spectra of Ub-T66C-DO3A recorded at various temperatures. Spectra were shifted horizontally relative to the 16 K one for clarity. **(B)** Calculated populations of electron spin levels $n(m_S)$ (upper panel) and electron spin transition polarizations $n(m_S)-n(m_S+1)$ (lower panels) at various temperatures. Populations were calculated as $n(m_S)=\exp[m_S h\nu/kT]/\sum_{m_S'}\exp[m_S' h\nu/kT]$, with ν=95 GHz, h – the Planck constant, k – the Boltzmann constant, T – temperature. **(C)** Experimental ED-EPR spectra at representative temperatures (colored lines) and their joint numerical simulation (orange lines) using an identical set of ZFS parameters (**Table S3**). **(D)** The experimental ED-EPR spectrum at 2K (black trace), its simulation (red lines) and contributions of the individual EPR transitions (colored lines).

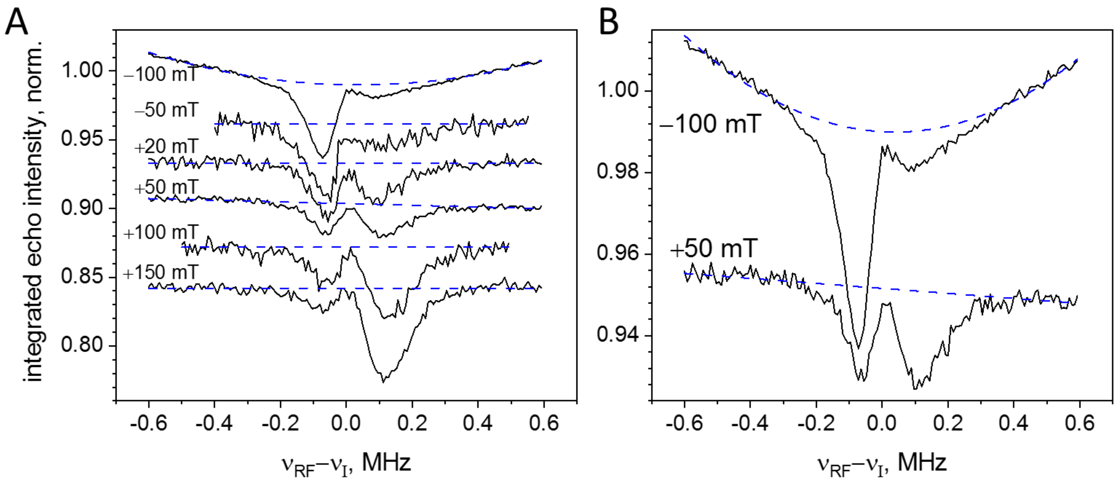

Fig. S10. Experimental ^{19}F Mims ENDOR spectra of Ub-T66C-DO3A recorded at 2K at different field positions relative to the CT (black traces) and the estimated baselines (blue dashed lines). At CT–100 mT, the baseline was approximated with a quadratic parabola and at CT+50 mT with a linear function; these baseline shapes are either due to the far lying ^{1}H ENDOR signals or to instrumental artifacts. At other field positions the background was taken as an horizontal straight line. In **(A)** all spectra are shown while in **(B)** the spectra for which the non-uniform background was subtracted are enlarged. The spectra are shifted for clarity in a vertical direction.

S10. Orientation selectivity in ^{19}F ENDOR of Ub-T66C-DO3A

The shape of the EPR spectrum $I\left(B_0\right)$ is a sum of contributions from various orientations:

$$I\left(B_0\right)=\int_{\theta=0}^{\pi}\sin\theta d\theta\int_{\varphi=0}^{2\pi}d\varphi\cdot F\left(B_0;\theta,\varphi\right) \quad \text{(S9)}$$

with θ and φ as polar and azimuthal angles that determine the orientation B_0 in the molecular (ZFS) frame and $F\left(B_0;\theta,\varphi\right)$ is the contribution of a given orientation $\left(\theta,\varphi\right)$ to the overall spectrum (note that F includes contribution of all ZFS parameters according to their distributions). Functions P_X, P_Y and P_Z represent the projection of the spectrum on X, Y and Z axes of the ZFS. These functions are defined as:

$$P_{X,Y,Z}\left(B_0\right)=\int_{\theta=0}^{\pi}\sin\theta d\theta\int_{\varphi=0}^{2\pi}d\varphi\cdot F\left(B_0;\theta,\varphi\right)\cdot a_{X,Y,Z}, \quad \text{(S10)}$$

with $a_X=\cos\varphi\sin\theta, a_Y=\sin\varphi\sin\theta, a_Z=\cos\theta$ the direction cosines of the B_0 vector, in the X, Y and Z frame. P_X, P_Y and P_Z reflect how many orientations close to the X,Y and Z axes are excited at a given magnetic field position. The simulated spectra of P_X, P_Y and P_Z are presented alongside the experimental and simulated ED-EPR spectra of the protein in **Fig. S11**. As can be appreciated, almost all orientations are excited at all field positions throughout the spectrum owing to the ZFS distribution. Nevertheless, the X orientations are excited with a somewhat larger probability in the vicinity of the CT, the Y orientations are slightly more dominant to the right of the CT, and the Z orientations are more pronounced to the left of the CT. Another way to illustrate the orientation contribution at each field position is presented in **Fig. S12**, where the probability of excitation at different orientations is shown for different field positions as a heat-plot on a unitary sphere.

Field dependent orientation contributions from the ED-EPR spectra simulations (**Figs. S11** and **S12**) were used to simulate the ENDOR spectra shown in **Fig. 4A** (main

text). In these simulations, the hyperfine splitting $a_\perp$, ENDOR line width and angles (θ_F, φ_F) that define the orientation of Gd–F dipolar vector were varied globally for all spectra, and the best fit values obtained were $a_\perp = 30.4\,\text{kHz}$, $\Delta_L = 36.1\,\text{kHz}$, $\Delta_G = 0$, $\theta_F = 86°, \varphi_F = 90°$, indicating that the Gd–F vector is directed close to the Y axis of the Gd(III) ZFS. Note that only the $|-7/2\rangle \leftrightarrow |-5/2\rangle$ EPR transition was considered to simulate the ENDOR spectra recorded at 2 K.

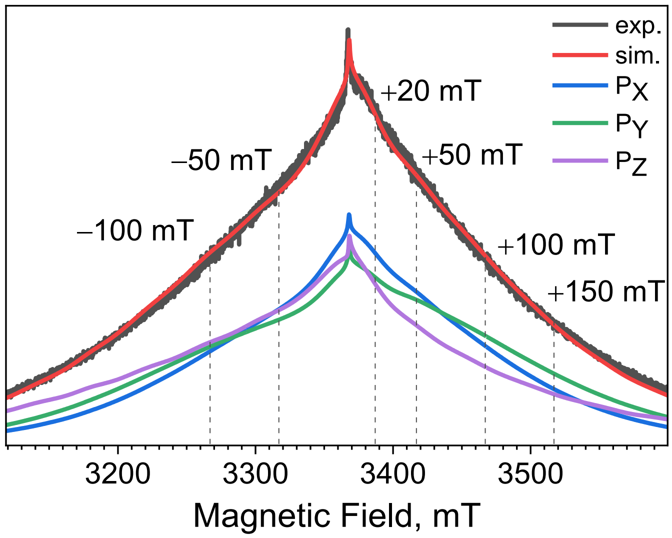

Fig. S11. ED-EPR spectrum of Ub-T66C-DO3A recorded at 2.2 K (black trace) and simulations (red lines). Blue, green and purple lines are the projections of the spectrum on the X, Y and Z axes of ZFS frame, illustrating the contributions of different orientations of the Gd complex ZFS tensor with respect to the static magnetic field.

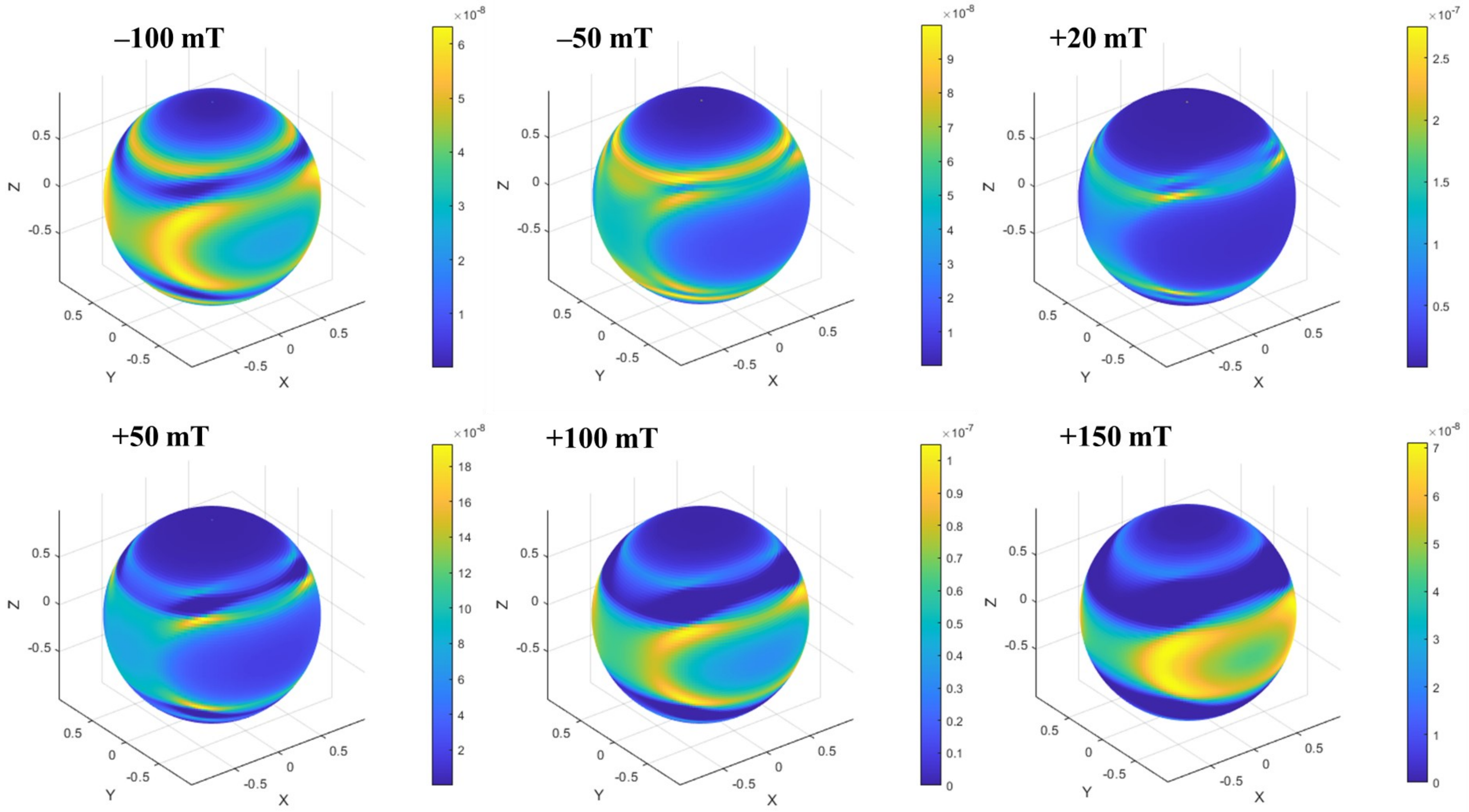

Fig. S12. Heat-plot representation of orientations (in the framework of the ZFS tensor of Gd(III)) of Ub-T66C-DO3A excited at given field positions (with respect to central transition) obtained from simulations of ED-EPR spectrum recorded at 2 K; only $|-7/2\rangle \leftrightarrow |-5/2\rangle$ EPR transition was considered.

Another approach to simulate the observed orientation selection behavior is to use the function $\rho(\beta; B_0)$, which provides for each field position the number density of molecules with orientation β between B_0 and the Gd(III)–^{19}F vector, parametrized by a truncated series of Legendre polynomials $P_n(\cos\beta)$:

$$\rho(\beta) = \max\left\{1 + \sum_{n=2,4,\ldots}^{L_{\max}} (2n+1) A_n P_n(\cos\beta), 0\right\} \quad \text{(S11)}$$

Truncating the series at $L_{\max} = 4$ is sufficient to describe the spectra. The best fit parameters A_2 and A_4 that define the orientation selection function at each field are listed in **Table S5** and the shapes of functions themselves are presented in **Fig. S13**.

Table S5. Expansion parameters A_2 and A_4 that define the orientation selection function $\rho(\beta)$ according to eq. (4).

	**–100 mT**	**–50 mT**	**+20 mT**	**+50 mT**	**+100 mT**	**+150 mT**
A_2	–0.265	–0.177	–0.051	0.015	0.118	0.439
A_4	0.063	0.019	–0.153	–0.100	–0.078	0.285

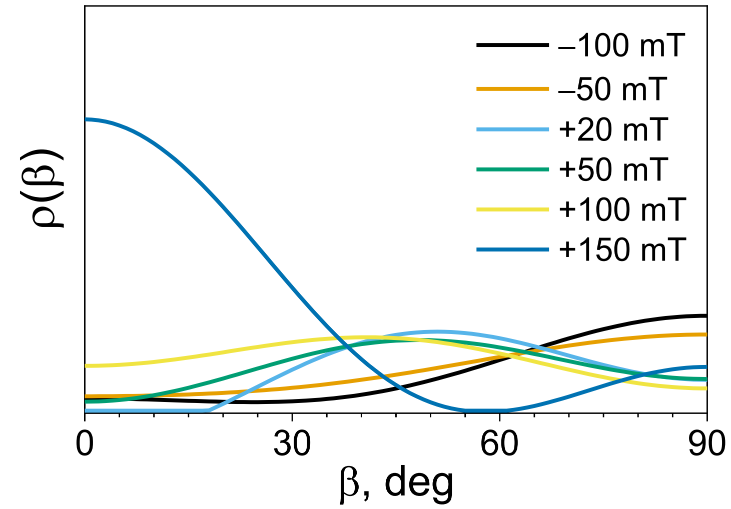

Fig. S13. Phenomenological orientation selection obtained from the simulations of the ENDOR spectra of Ub-T66C-DO3A using eq. S11.

S11. ^{19}F ENDOR spectra of Ub-T66C-DO3A recorded at 2.2 K and 6 K

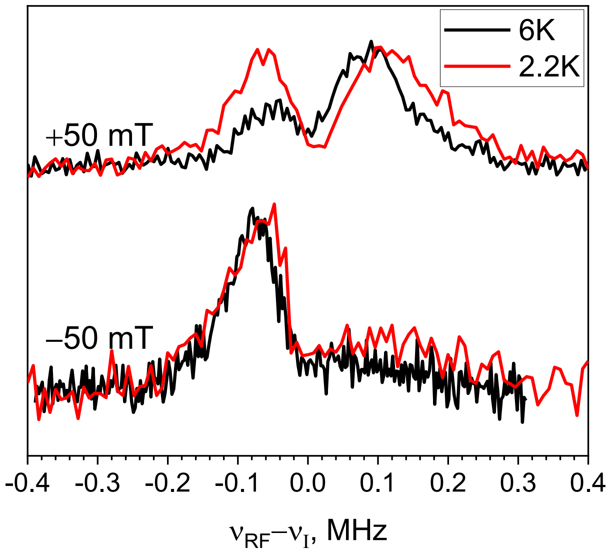

Fig. S14. Comparison of the ^{19}F Mims ENDOR spectra of Ub-T66C-DO3A recorded at 2.2 K (red traces) and 6 K (black traces). The difference between the two spectra at +50mT probably arises from the more pronounced contribution of $|-5/2\rangle \leftrightarrow |-3/2\rangle$ EPR transition.

S12. Supplementary data on GB1-Q32C-DO3A

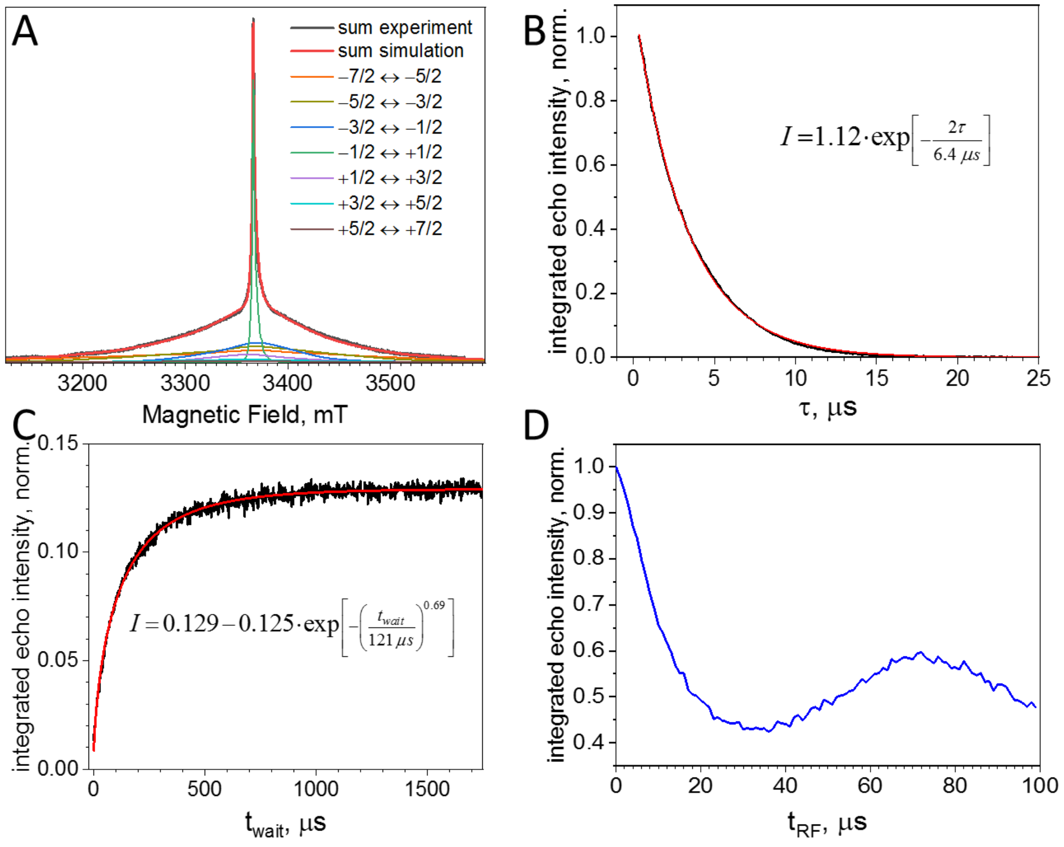

Fig. S15. (A) Experimental ED-EPR spectrum of GB1-Q32C-DO3A at 6 K (black trace) and simulation (red trace); contributions of individual EPR transitions are shown as colored lines. The spectrum was recorded using a Mims ENDOR echo sequence with τ=2 µs, and RF off-resonance with ^{1}H ENDOR lines, ν_{RF} = 136.4 MHz. The ZFS parameters used in the simulation are listed in **Table S3**. **(B)** Echo decay at CT and 6 K (black trace) and its exponential fit (red line). **(C)** Inversion recovery trace measured at CT at 6 K (black) and its stretched exponential fit (red line). **(D)** ^{1}H Rabi nutations measured by Mims ENDOR sequence with a variable RF pulse length; τ=550 ns, ν_{RF}=144.5 MHz.

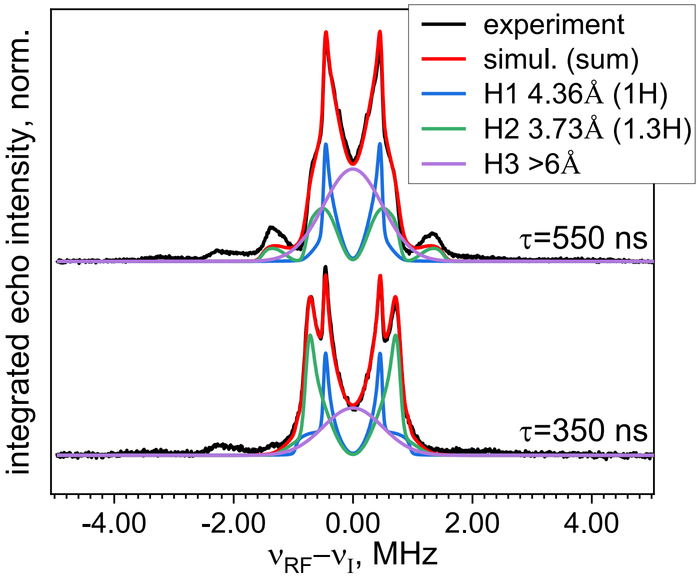

Fig. S16. Experimental Mims ^{1}H ENDOR spectra of GB1-Q32C-DO3A recorded at the CT with two τ values (black traces) and their numerical simulation (red trace). Colored lines show the individual contributions of different proton types. Gd–H distances and the relative number of proton types are listed in the figure.

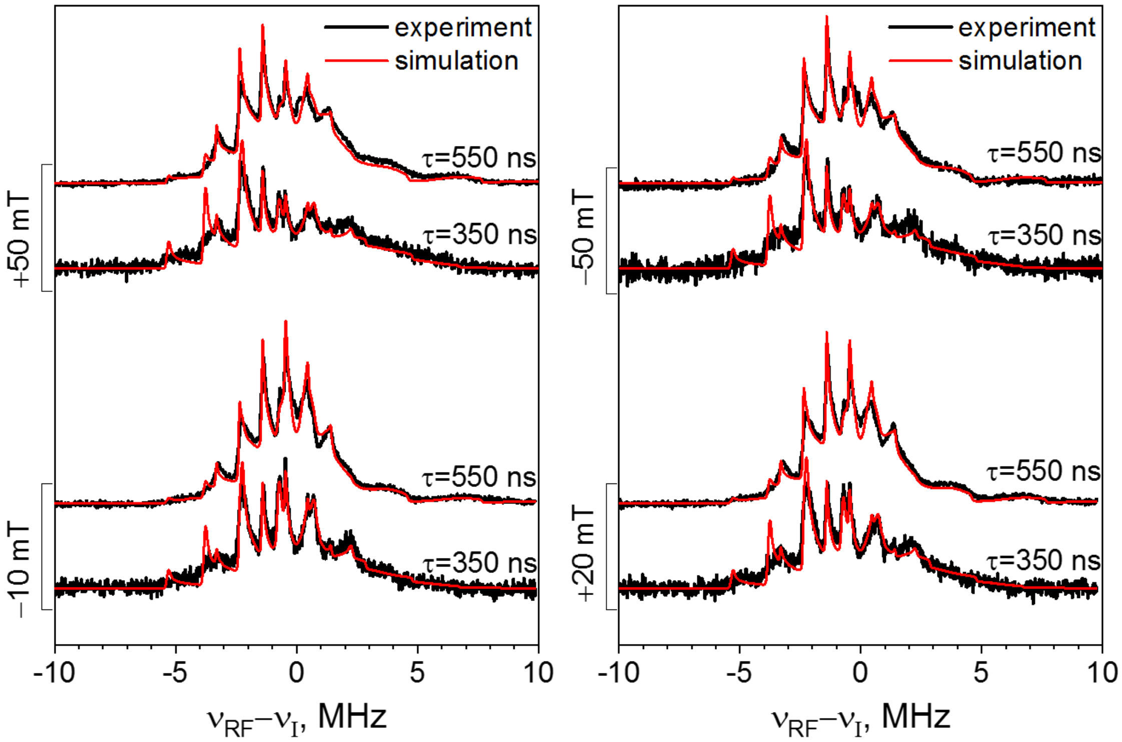

Fig. S17. Experimental Mims ^1H ENDOR spectra of GB1-Q32C-DO3A recorded at different positions off CT (black traces) and their simulation (red traces).

Table S6. Relative intensities of Gd(III) EPR transitions determined from simulations of the ^{1}H ENDOR spectra (6 K) of GB1-Q32C-DO3A (first value) and from the simulation of the ED-EPR (second value). The transition intensities are normalized to unity.

	**–50 mT**	**–10 mT**	**+20 mT**	**+50 mT**
$\lvert -7/2\rangle \leftrightarrow \lvert -5/2\rangle$	0.251 / 0.257	0.222 / 0.204	0.225 / 0.211	0.328 / 0.263
$\lvert -5/2\rangle \leftrightarrow \lvert -3/2\rangle$	0.501 / 0.305	0.382 / 0.269	0.454 / 0.275	0.454 / 0.329
$\lvert -3/2\rangle \leftrightarrow \lvert -1/2\rangle$	0.188 / 0.245	0.297 / 0.313	0.254 / 0.327	0.150 / 0.261
$\lvert -1/2\rangle \leftrightarrow \lvert +1/2\rangle$	0 / 0	0 / 0.022	0 / 0.028	0 / 0
$\lvert +1/2\rangle \leftrightarrow \lvert +3/2\rangle$	0.059 / 0.111	0.099 / 0.134	0.067 / 0.108	0.068 / 0.092
$\lvert +3/2\rangle \leftrightarrow \lvert +5/2\rangle$	0 / 0.060	0 / 0.044	0 / 0.038	0 / 0.041
$\lvert +5/2\rangle \leftrightarrow \lvert +7/2\rangle$	0 / 0.020	0 / 0.013	0 / 0.012	0 / 0.013

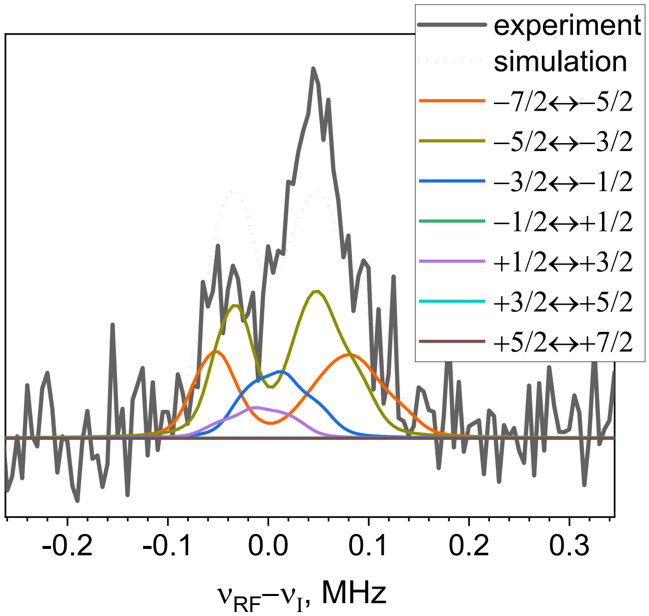

Fig. S18. Contributions of different EPR transitions to the ^{19}F Mims ENDOR spectrum of GB1-Q32C-DO3A, recorded at the position +50 mT from the CT; experimental spectrum (gray line), simulated spectrum (red dashed line), individual contributions of the EPR transitions (colored lines). Note the absence of the CT.

S13. Simulated ENDOR spectra for various Gd–F distances

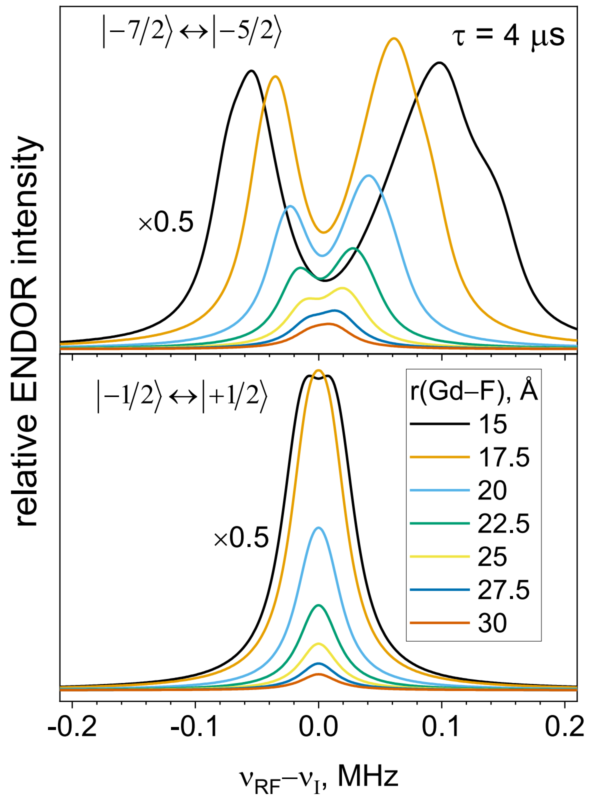

Fig. S19. Simulated ^{19}F ENDOR spectra illustrating the shape dependence of the spectra for the $|-7/2\rangle \leftrightarrow |-5/2\rangle$ (upper panel) and $|-1/2\rangle \leftrightarrow |+1/2\rangle$ (lower panel) EPR transitions on the Gd–F distance for purely Lorentzian lines with $\Delta_L = 20\,\text{kHz}$. The intensity of spectrum at the shortest distance is scaled by 0.5.

Supplementary References

(1) Laurent, S.; Vander Elst, L.; Galaup, C.; Leygue, N.; Boutry, S.; Picard, C.; Muller, R. N. Bifunctional Gd(III) and Tb(III) Chelates Based on a Pyridine–Bis(Iminodiacetate) Platform, Suitable Optical Probes and Contrast Agents for Magnetic Resonance Imaging. *Contrast Media & Molecular Imaging* **2014**, *9* (4), 300-312. DOI: 10.1002/cmmi.1576.

(2) Frisch, M. J.; Trucks, G. W.; Schlegel, H. B.; Scuseria, G. E.; Robb, M. A.; Cheeseman, J. R.; Scalmani, G.; Barone, V.; Petersson, G. A.; Nakatsuji, H.; Li, X.; Caricato, M.; Marenich, A. V.; Bloino, J.; Janesko, B. G.; Gomperts, R.; Mennucci, B.; Hratchian, H. P.; Ortiz, J. V.; Izmaylov, A. F.; Sonnenberg, J. L.; Williams-Young, D.; Ding, F.; Lipparini, F.; Egidi, F.; Goings, J.; Peng, B.; Petrone, A.; Henderson, T.; Ranasinghe, D.; Zakrzewski, V. G.; Gao, J.; Rega, N.; Zheng, G.; Liang, W.; Hada, M.; Ehara, M.; Toyota, K.; Fukuda, R.; Hasegawa, J.; Ishida, M.; Nakajima, T.; Honda, Y.; Kitao, O.; Nakai, H.; Vreven, T.; Throssell, K.; J. A. Montgomery, J.; Peralta, J. E.; Ogliaro, F.; Bearpark, M. J.; Heyd, J. J.; Brothers, E. N.; Kudin, K. N.; Staroverov, V. N.; Keith, T. A.; Kobayashi, R.; Normand, J.; Raghavachari, K.; Rendell, A. P.; Burant, J. C.; Iyengar, S. S.; Tomasi, J.; Cossi, M.; Millam, J. M.; Klene, M.; Adamo, C.; Cammi, R.; Ochterski, J. W.; Martin, R. L.; Morokuma, K.; Farkas, O.; Foresman, J. B.; Fox, D. J. *Gaussian 16, Revision C.01*; Gaussian, Inc.: Wallingford CT, 2016.

(3) Perdew, J. P.; Burke, K.; Ernzerhof, M. Generalized Gradient Approximation Made Simple. *Phys. Rev. Lett.* **1996**, *77* (18), 3865-3868. DOI: 10.1103/PhysRevLett.77.3865.

(4) Perdew, J. P.; Burke, K.; Ernzerhof, M. Gerneralized Gradient Approximation Made Simple [Phys. Rev. Lett. 77, 3865 (1996)]. *Phys. Rev. Lett.* **1997**, *78* (7), 1396, Erratum. DOI: 10.1103/PhysRevLett.78.1396.

(5) Grimme, S.; Antony, J.; Ehrlich, S.; Kreig, H. A Consistent and Accurate Ab Initio Parametrization of Density Functional Dispersion Correction (Dft-D) for the 94 Elements H-Pu. *J. Chem. Phys.* **2010**, *132* (15), 154104, Article. DOI: 10.1063/1.3382344.

(6) Grimme, S.; Ehrlich, S.; Goerigk, L. Effect of the Damping Function in Dispersion Corrected Density Functional Theory. *J. Comput. Chem.* **2011**, *32* (7), 1456-1465, Article. DOI: 10.1002/jcc.21759.

(7) Weigend, F. Accurate Coulomb-Fitting Basis Sets for H to Rn. *Phys. Chem. Chem. Phys.* **2006**, *8*, 1057-1065, Article. DOI: 10.1039/b515623h.

(8) Weigend, F.; Ahlrichs, R. Balanced Basis Sets of Split Valence, Triple Zeta Valence and Quadruple Zeta Valence Quality for H to Rn: Design and Assessment of Accuracy. *Phys. Chem. Chem. Phys.* **2005**, *7*, 3297-3305, Article. DOI: 10.1039/B508541A.

(9) Dunlap, B. I. Fitting the Coulomb Potential Variationally in *X*α Molecular Calculations. *J. Chem. Phys.* **1983**, *78* (6), 3140-3142, Article. DOI: 10.1063/1.445228.

(10) Dunlap, B. I. Robust and Variational Fitting: Removing the Four-Center Integrals from Center Stage in Quantum Chemistry. *Journal of Molecular Structure: THEOCHEM* **2000**, *529* (1-3), 37-40, Article. DOI: 10.1016/S0166-1280(00)00528-5

(11) Dolg, M.; Stoll, H.; Preuss, H. Energy-Adjusted *Ab Initio* Pseudopotentials for the Rare Earth Elements. *J. Chem. Phys.* **1989**, *90* (3), 1730-1734, Article. DOI: 10.1063/1.456066.

(12) Gulde, R.; Pollak, P.; Weigend, F. Error-Balanced Segmented Contracted Basis Sets of Double-Z to Quadruple-Z Valence Quality for the Lanthanides. *Journal of Chemical Theory and Computation* **2012**, *8* (11), 4062-4068, Article. DOI: 10.1021/ct300302u.

(13) Pritchard, B. P.; Altarawy, D.; Didier, B.; Gibson, T. D.; Windus, T. L. New Basis Set Exchange: An Open, up-to-Date Resource for the Molecular Sciences Community. *Journal of Chemical Information and Modeling* **2019**, *59* (11), 4814-4820, Article. DOI: 10.1021/acs.jcim.9b00725.

(14) Feller, D. The Role of Databases in Support of Computational Chemistry Calculations. *J. Comput. Chem.* **1996**, *17* (13), 1571-1586, Article. DOI: 10.1002/(SICI)1096-987X(199610)17:13<1571::AID-JCC9>3.0.CO;2-P.

(15) Schuchardt, K. L.; Didier, B. T.; Elsethagen, T.; Sun, L.; Gurumoorthi, V.; Chase, J.; Li, J.; Windus, T. L. Basis Set Exchange: A Community Database for Computational Sciences. *Journal of Chemical Information and Modeling* **2007**, *47* (3), 1045-1052. DOI: 10.1021/ci600510j.

(16) Marenich, A. V.; Cramer, C. J.; Truhlar, D. G. Universal Solvation Model Based on Solute Electron Density and on a Continuum Model of the Solvent Defined by

the Bulk Dielectric Constant and Atomic Surface Tensions. *J. Phys. Chem. B* **2009**, *113* (18), 6378-6396, Article. DOI: 10.1021/jp810292n.

(17) Zhao, Y.; Truhlar, D. G. Design of Density Functionals That Are Broadly Accurate for Thermochemistry, Thermochemical Kinetics, and Nonbonded Interactions. *J. Phys. Chem. A* **2005**, *109* (25), 5656-5667, Article. DOI: 10.1021/jp050536c.

(18) Burke, K.; Perdew, J. P.; Wang, Y. Electronic Density Functional Theory: Recent Progress and New Directions. In *Electronic Density Functional Theory: Recent Progress and New Directions*, Dobson, J. F., Vignale, G., Das, M. P. Eds.; Plenum Publishing, 1998.

(19) Perdew, J. P. Electronic Structures of Solids. In *Electronic Structures of Solids*, Ziesche, P., Eschrig, H. Eds.; Akademie Verlag, 1991; p 11.

(20) Perdew, J. P.; Burke, K.; Wang, Y. Generalized Gradient Approximation for the Exchange-Correlation Hole of a Many-Electron System. *Phys. Rev. B* **1996**, *54* (23), 16533-16539. DOI: 10.1103/PhysRevB.54.16533.

(21) Perdew, J. P.; Chevary, J. A.; Vosko, S. H.; Jackson, K. A.; Pederson, M. A.; Singh, D. J.; Fiolhais, C. Atoms, Molecules, Solids, and Surfaces: Applications of the Generalized Gradient Approximation for Exchange and Correlation. *Phys. Rev. B* **1992**, *46* (11), 6671-6687.

(22) Perdew, J. P.; Chevary, J. A.; Vosko, S. H.; Jackson, K. A.; Pederson, M. A.; Singh, D. J.; Fiolhais, C. Erratum: Atoms, Molecules, Solids, and Surfaces: Applications of the Generalized Gradient Approximation for Exchange and Correlation. *Phys. Rev. B* **1993**, *48* (7), 4978, Erratum. DOI: 10.1103/Phys.Rev.B.48.4978.2.

(23) Becke, A. D. Density-Functional Thermochemistry. Iv. A New Dynamical Correlation Functional and Implications for Exact-Exchange Mixing. *J. Chem. Phys.* **1996**, *104* (3), 1040-1046, Article. DOI: 10.1063/1.470829.

(24) Caldeweyher, E.; Bannwarth, C.; Grimme, S. Extension of the D3 Dispersion Coefficient Model. *J. Chem. Phys.* **2017**, *147* (3), 034112, Article. DOI: 10.1063/1.4993215.

(25) Caldeweyher, E.; Ehlert, S.; Hansen, A.; Neugebauer, H.; Spicher, S.; Bannwarth, C.; Grimme, S. A Generally Applicable Atomic-Charge Dependent London

Dispersion Correction. *J. Chem. Phys.* **2019**, *150* (15), 154122, Article. DOI: 10.1063/1.5090222.

(26) *D4 - a Generally Applicable Atomic-Charge Dependent London Dispersion Correction*. https://www.chemie.uni-bonn.de/grimme/de/software/dft-d4 (accessed 2024-02-02).

(27) Baranyai, Z.; Tei, L.; Giovenzana, G. B.; Kálmán, F. K.; Botta, M. Equilibrium and NMR Relaxometric Studies on the S-Triazine-Based Heptadentate Ligand Ptdita Showing High Selectivity for Gd^{3+} Ions. *Inorg. Chem.* **2012**, *51* (4), 2597-2607. DOI: 10.1021/ic202559h.

(28) Pellegatti, L.; Zhang, J.; Drahos, B.; Villette, S.; Suzenet, F.; Guillaumet, G.; Petoud, S.; Tóth, É. Pyridine-Based Lanthanidecomplexes: Towards Bimodal Agents Operating as near Infrared Luminescent and MRI Reporters. *Chemical Communications* **2008**, (48), 6591-6593. DOI: 10.1039/B817343E.

(29) Feintuch, A.; Shimon, D.; Hovav, Y.; Banerjee, D.; Kaminker, I.; Lipkin, Y.; Zibzener, K.; Epel, B.; Vega, S.; Goldfarb, D. A Dynamic Nuclear Polarization Spectrometer at 95 GHz / 144 MHz with EPR and NMR Excitation and Detection Capabilities. *J. Magn. Reson.* **2011**, *209* (2), 136-141. DOI: 10.1016/j.jmr.2010.12.010.

(30) Gromov, I.; Krymov, V.; Manikandan, P.; Arieli, D.; Goldfarb, D. A W-Band Pulsed ENDOR Spectrometer: Setup and Application to Transition Metal Centers. *J. Magn. Reson.* **1999**, *139* (1), 8-17. DOI: 10.1006/jmre.1999.1762.

(31) Mentink-Vigier, F.; Collauto, A.; Feintuch, A.; Kaminker, I.; Tarle, V.; Goldfarb, D. Increasing Sensitivity of Pulse EPR Experiments Using Echo Train Detection Schemes. *J. Magn. Reson.* **2013**, *236*, 117-125. DOI: 10.1016/j.jmr.2013.08.012.

(32) Epel, B.; Arieli, D.; Baute, D.; Goldfarb, D. Improving W-Band Pulsed ENDOR Sensitivity—Random Acquisition and Pulsed Special Triple. *J. Magn. Reson.* **2003**, *164* (1), 78-83. DOI: 10.1016/S1090-7807(03)00191-5.

(33) Raitsimring, A.; Astashkin, A. V.; Enemark, J. H.; Kaminker, I.; Goldfarb, D.; Walter, E. D.; Song, Y.; Meade, T. J. Optimization of Pulsed-DEER Measurements for Gd-Based Labels: Choice of Operational Frequencies, Pulse Durations and Positions, and Temperature. *Applied Magnetic Resonance* **2013**, *44* (6), 649-670. DOI: 10.1007/s00723-012-0434-6.

(34) Raitsimring, A. M.; Astashkin, A. V.; Poluektov, O. G.; Caravan, P. High-Field Pulsed EPR and ENDOR of Gd^{3+} Complexes in Glassy Solutions. *Applied Magnetic Resonance* **2005**, *28* (3), 281-295. DOI: 10.1007/BF03166762.

(35) Clayton, J. A.; Keller, K.; Qi, M.; Wegner, J.; Koch, V.; Hintz, H.; Godt, A.; Han, S.; Jeschke, G.; Sherwin, M. S.; Yulikov, M. Quantitative Analysis of Zero-Field Splitting Parameter Distributions in Gd(III) Complexes. *Phys. Chem. Chem. Phys.* **2018**, *20* (15), 10470-10492. DOI: 10.1039/C7CP08507A.

(36) Dennis, J. E.; Gay, D. M.; Walsh, R. E. An Adaptive Nonlinear Least-Squares Algorithm. *ACM Trans. Math. Softw.* **1981**, *7* (3), 348–368. DOI: 10.1145/355958.355965.
